# Scoping review of interventions aimed at promoting healthy screen use among adolescents

**DOI:** 10.1136/bmjopen-2025-103772

**Published:** 2025-09-21

**Authors:** Wing Lam Tock, Lise Gauvin, Frédérique Hudon, Frédérique Tremblay, Richard E Bélanger, Anne-Marie Turcotte-Tremblay

**Affiliations:** 1 Department of Social and Preventive Medicine, École de santé publique, Université de Montréal, Montréal, Québec, Canada; 2 Centre de Recherche du Centre Hospitalier de l’Universite de Montreal, Montréal, Québec, Canada; 3 Faculté de médecine, Université Laval, Québec, Québec, Canada; 4 VITAM Centre de Recherche en Santé Durable, Québec, Québec, Canada; 5 Faculté des sciences infirmières, Université Laval, Québec, Québec, Canada

**Keywords:** Adolescents, PUBLIC HEALTH, Systematic Review, Behavior

## Abstract

**Abstract:**

**Objectives:**

The objective of this scoping review is to map and synthesise existing literature on interventions aimed at promoting healthy screen use among adolescents. This review identifies the types, functions and settings of interventions, explores the diversity of targeted outcomes and highlights equity considerations and research gaps.

**Design:**

We conducted a scoping review in accordance with the Arksey and O’Malley framework and reported following the Preferred Reporting Items for Systematic Reviews and Meta-Analyses Extension for Scoping Reviews guidelines.

**Data sources:**

We systematically searched Medline, PsycINFO and ERIC from January 2013 to June 2024. Reference lists of included studies were also manually screened.

**Eligibility criteria:**

We included peer-reviewed experimental, quasi-experimental, observational and qualitative studies reporting on interventions designed to promote healthy screen use among adolescents aged 10–19 years.

**Data extraction and synthesis:**

One author extracted data using a structured charting form, and a second author verified all entries. Results were synthesised descriptively across key themes including target populations, theoretical frameworks, intervention components and reported outcomes.

**Results:**

From 6433 records, we identified 93 articles on 81 interventions, mainly conducted in high school settings in the USA and Australia. Most examined short-term interventions targeting recreational screen time. Outcomes included media literacy, cyberbullying, internet and gaming addiction, safe internet use, social media use and mental and sexual health. Seventy-eight per cent of interventions attempted to educate adolescents, while 34% offered training activities (eg, educational sessions to elevate risk awareness and skill-based training to enhance digital literacy and self-regulation). Interventions targeting external influences were used less frequently. About 20% of studies showed no statistically significant findings, highlighting the need to promote evidence-based interventions.

**Conclusion:**

This review identifies a need for broader, multilevel strategies that account for contextual factors and social determinants in influencing screen use and its related health issues. Future research should explore long-term effectiveness while examining the potential moderating and mediating effects of social determinants. Equity considerations were not a primary focus of most interventions, underscoring an important gap in this literature. Future interventions could incorporate equity-focused design and evaluation to ensure they respond to the needs of diverse adolescent populations.

**STRENGTHS AND LIMITATIONS OF THIS STUDY**

- The scoping review adheres to the established Arksey and O'Malley framework for conducting a scoping review, ensuring transparency and reproducibility in the study process.
- The inclusion of diverse study designs (experimental, quasi-experimental, observational, mixed, qualitative) strengthens the generalisability and validity of the findings.
- The review adopts a robust theoretical framework (the intervention functions outlined by Michie *et al*) to classify interventions, providing a systematic approach to understanding the range of intervention strategies.
- Grey literature and non-empirical studies were excluded, which may have provided additional insights into intervention design and implementation processes.
- The exclusion of studies targeting adolescents with specific health conditions, such as obesity, may limit the applicability of findings to at-risk populations.

## Introduction

 Adolescents are increasingly immersed in screens due to the rise of social media, online entertainment and instant communication, all reinforced by constant connectivity and easy access to digital devices.^1^ Screen-based digital media can yield meaningful benefits for adolescents, especially when usage is moderate and purposeful. For instance, social media can enhance social connectedness and belonging by enabling peer support networks, identity exploration and creative expression.^2^ Yet, some digital platforms are deliberately designed to capture and sustain attention through features such as infinite scrolling, algorithmic personalisation and notifications on social media, or randomised reward systems such as loot boxes in video games.^3^ These features are thought to create feedback loops that encourage habituation and excessive engagement by triggering the release of dopamine, a neurotransmitter that reinforces rewarding behaviours.^4^

The widespread engagement with screens has prompted consideration of its consequences on the mental and physical health of young people.^5^ Research indicates that excessive screen time is associated with a range of negative outcomes, including elevated metabolic health risk,^6^ anxiety and depression,^7^ and poor academic performance.^8^ Moreover, adolescents who engage excessively in screen activities are often less likely to adhere to physical activity guidelines.^9^ Evidence increasingly shows that sedentary behaviours, including extended periods spent watching television and other screen media, may contribute to heightened risk of cardiometabolic diseases later in life.^10^ Furthermore, screen-related habits such as compulsive internet use, gaming addiction and cyberbullying are becoming more prevalent.^11^ For example, a systematic review of 63 studies revealed rates of cyberbullying ranging from 13.99% to 57.5%.^12^ A meta-analysis of 16 studies on gaming disorder prevalence in adolescents showed an overall prevalence of 4.6% (6.8% for males and 1.3% for females).^13^ This emerging evidence presents new challenges for public health practitioners who must adapt to rapidly evolving technologies while promoting healthy screen-related behaviours among youth.^14^ Furthermore, social determinants of health such as neighbourhood safety and access to recreational space could influence how adolescents engage with screens. Recent studies show that limited access to safe outdoor environments is associated with higher screen use, while greater exposure to green space is linked to lower recreational screen time and better mental health.^15^ Recognising these structural influences reinforces the need for multilevel intervention approaches that address both individual behaviours and environmental contexts.

In response to these challenges, practitioners involved in public health, schools and community organisations are developing initiatives to promote healthy screen use and prevent problematic use among adolescents.^16^ Digital wellness workshops and prevention programmes are increasingly being offered in schools.^17^ These interventions focus on reducing internet addiction,^18 19^ cyberbullying^20^ and sexting^21^ as well as on developing critical judgement by promoting knowledge, skills and attitudes towards risky behaviours.^22^ Some interventions focus on motivational/volitional changes,^23^ while others prioritise modifying physical environments or policies.^24^ According to Jones and colleagues, behavioural interventions that include techniques such as time reduction goal setting, goal revision and self-monitoring can have positive effects.^16^ However, there is no consensus on which strategies should be prioritised, at school or at home, to promote healthy screen use among adolescents.

There remains a significant gap in synthesising evidence to fully capture the evolving scope of this topic and identify research priorities. The existing body of research on screen time interventions is fragmented and often inconclusive, primarily due to the diversity of interventions and methodological shortcomings (eg, small sample size, short follow-up period).^16 25^ Current reviews of interventions targeting screen-use behaviours among adolescents tend to focus narrowly on single outcomes such as screen time,^26^,^28^ media literacy or cyberbullying.^29^ Most existing reviews focus on quantifying intervention effects, while excluding qualitative studies that explore stakeholder perceptions and implementation processes.^26^ Moreover, previous reviews may not fully reflect most recent developments, as they do not capture interventions tested in the past 5 years in response to rapid technological advancements that transform how adolescents interact with digital screens. Furthermore, the concept of ‘healthy screen use’ remains inconsistently defined and operationalised across the literature. Multiple overlapping concepts such as ‘digital wellness’, ‘balanced screen use’ and ‘healthy media’ are used in adolescent screen research, but none has achieved widespread consensus.^30^ For instance, digital wellness frameworks emphasise mindful engagement rather than the absence of harm,^31^ while 24-hour movement models distinguish screen time by context, such as educational vs recreational use.^32^

These limitations make it difficult for school and community stakeholders to decide which interventions to implement and how. An updated, comprehensive synthesis that maps the extent and types of evidence-related interventions targeting behaviours related to healthy screen use is lacking. This scoping review synthesises existing literature on interventions aimed at promoting healthy screen use among adolescents, while addressing the populations, settings, study designs, intervention types, key concepts and theoretical frameworks mobilised, in order to identify methodological trends, areas of consensus and disagreement, limitations and research gaps.

## Methods

### Protocol and registration

We conducted a scoping review to map key concepts and the main sources and types of evidence available.^33^ We followed Arksey and O’Malley’s methodological approach to promote accuracy and reproducibility^34^ and respected Preferred Reporting Items for Systematic Reviews and Meta-Analyses Extension for Scoping Reviews (PRISMA-ScR) guidelines.^35^ The protocol was registered through the Open Science Framework (OSF) platform (https://doi.org/10.17605/OSF.IO/CP8BH).

### Inclusion and exclusion criteria

Table 1 summarises the inclusion and exclusion criteria. This review included articles published from January 2013 to June 2024. The 2013 starting point was chosen because it coincides with changes in screen technology, the rise of smartphones, social media platforms (eg, TikTok, Snapchat), algorithm-driven engagement and faster mobile networks (4G LTE).^36^ Earlier articles may not be relevant to current screen-related behaviours among adolescents.

**Table 1:** Inclusion and exclusion criteria for studies

	Inclusion criteria	Exclusion criteria
Dates	2013–2024	Before 2013
Language	All languages	None
Country	All countries	None
Publication type	Peer-reviewed articles presenting empirical data	Protocols, editorials, guidelines, commentaries
Study type	Experimental, quasi-experimental or observational studies including qualitative, quantitative or mixed methods	No design or methods presented No empirical evidence presented
Population (P)	Adolescents 10–19 years (studies with a subsample of adolescents between 10 and 19 years were included), parents of adolescents, or individuals involved in their education (eg, educators, health professionals, law enforcement representatives)	Adolescents below 10 years or above 19 years Population studied consisted solely of adolescents with specific health conditions, such as obesity (BMI>95 th percentile) or video game addiction
Intervention type (I)	Interventions or prevention programmes of any duration, frequency, delivery or components designed to promote healthy screen use	Interventions not designed to promote healthy screen use as a primary or secondary objective
Comparison (C)	No restrictions concerning comparison	
Outcomes (O)	Any primary and secondary outcomes related to healthy screen use	No presentation of results related to screen use

BMI, body mass index.

Eligibility criteria followed the population, intervention, comparison, outcomes strategy (table 1). Adolescents, defined by the WHO as those aged 10–19 years, were the primary targeted study population.^37^ Interventions targeting parents of adolescents, educators, health professionals or law enforcement representatives were included when the intervention’s primary aim was to promote healthy screen use among adolescents, recognising that these adult stakeholders play a key role in shaping adolescent screen-related behaviours and environments. Studies were also eligible if they included a broader age range but reported outcomes relevant to a subsample of participants aged 10–19 years. Studies were excluded if they focused on adolescents with specific health conditions, such as obesity (body mass index (BMI)>95th percentile) or video game addiction. Articles included had to evaluate interventions designed to promote healthy screen use. Interventions were defined as structured sets of actions designed to influence the expected development of a phenomenon within a specific environment and timeframe, with the goal of addressing and ameliorating a problematic situation.^38 39^ Such interventions could encompass strategies that support individuals in achieving a healthier digital life balance, educational programmes to foster digital literacy, guidelines for parents on screen time management and tools for self-regulation of digital habits. The intervention goals had to be focused on enhancing adolescents’ ability to use digital technology in a way that supports personal development and well-being, rather than detracting from it.^40^

There were no restrictions concerning comparison. Outcomes of interest were related to healthy use of screens. Based on the literature, the concept ‘healthy screen use’ refers to engaging with digital content that contributes positively to one’s well-being, encouraging active rather than passive consumption.^40^ Healthy screen use also designates intentional and balanced engagement with digital screens to maximise benefits and minimise harms associated with physical, mental and social well-being. This concept emphasises managing screen time effectively, choosing high-quality content and ensuring digital activities do not adversely impact essential aspects of life such as sleep, physical activity or face-to-face social interactions.^41^

### Information sources

Three databases, Medline (Ovid), PsycInfo (Ovid) and ERIC (Ovid) were searched. We also manually searched the reference lists of included studies to identify additional relevant articles.

### Search strategy

With the information specialist, we developed a list of Medical Subject Headings (MeSH) terms and keywords for concepts related to ‘adolescents’, ‘screen usage’ and ‘interventions’ (see online supplemental material 1: Full search strategies).

### Selection of evidence sources

Articles were screened using Covidence.^42^ References from the search strategy were imported into Covidence with article abstracts. Duplicates were automatically removed. Two reviewers independently conducted a two-step review process (title/abstract, full text). They judged titles and abstracts of potentially relevant studies based on the inclusion and exclusion criteria (table 1) and searched for relevant publications in reference lists. Disagreements between reviewers were resolved via discussion. For both steps of the review process (title/abstract, full text), 10% of articles were screened by two reviewers to ensure consistency.^43^ When disagreements persisted, a third researcher was brought into the discussion.

### Data extraction and charting

We developed a data extraction form using Microsoft Excel. One author (FH) performed data extraction, and a second author (WLT) verified all entries. Disagreements were resolved through discussion. When disagreements persisted, a third author was called in. The following information was extracted to summarise the articles: authors, title, year of publication, study location (country), study design, setting, aims, intervention name, target populations (sample size, age), theoretical and conceptual frameworks, programme evaluation approach, intervention function, intervention description, duration, intervention delivery, eligibility criteria, assessment timepoint, outcomes and results. We grouped countries by income level (ie, high, upper middle, lower middle, low) using World Bank’s classification.^44^ We also adopted Michie and colleagues’ framework to classify interventions in terms of their functions.^39^ This framework details nine distinct intervention functions*:*

1. Education: increasing knowledge or understanding.
2. Persuasion: using communication to induce positive or negative feelings or stimulate action.
3. Incentivisation: creating expectation of reward.
4. Training: imparting skills.
5. Enablement: increasing means/reducing barriers to increase capability or opportunity. (Capability beyond education and training; opportunity beyond environmental restructuring.)
6. Coercion: creating expectation of punishment or cost.
7. Restriction: using rules to reduce the opportunity to engage in the target behaviour (or to increase the target behaviour by reducing the opportunity to engage in competing behaviours).
8. Environmental restructuring: changing the physical or social context.
9. Modelling: providing an example for people to aspire to or imitate.^39^^(p7, table 1)^

### Data synthesis

To map the available evidence, we adopted a descriptive synthesis approach.^45^ This allowed for a broad perspective on available evidence and ensured that the synthesis captured the diversity of interventions being implemented worldwide. The extracted data included in this review were synthesised to cover the following themes:

- Characteristics of the included articles.
- Populations targeted.
- Theoretical and conceptual frameworks mobilised.
- Evaluation approaches used.
- Functions of the included interventions.
- Duration of interventions and studies’ follow-up periods.
- Outcomes targeted by the interventions.
- Key findings.
- Lessons learned from studying intervention implementation processes.
- Considerations for equity or differing effects based on population subgroups.

### Patient and public involvement statement

There was no patient or public involvement in this study.

## Results

**Figure 1:**
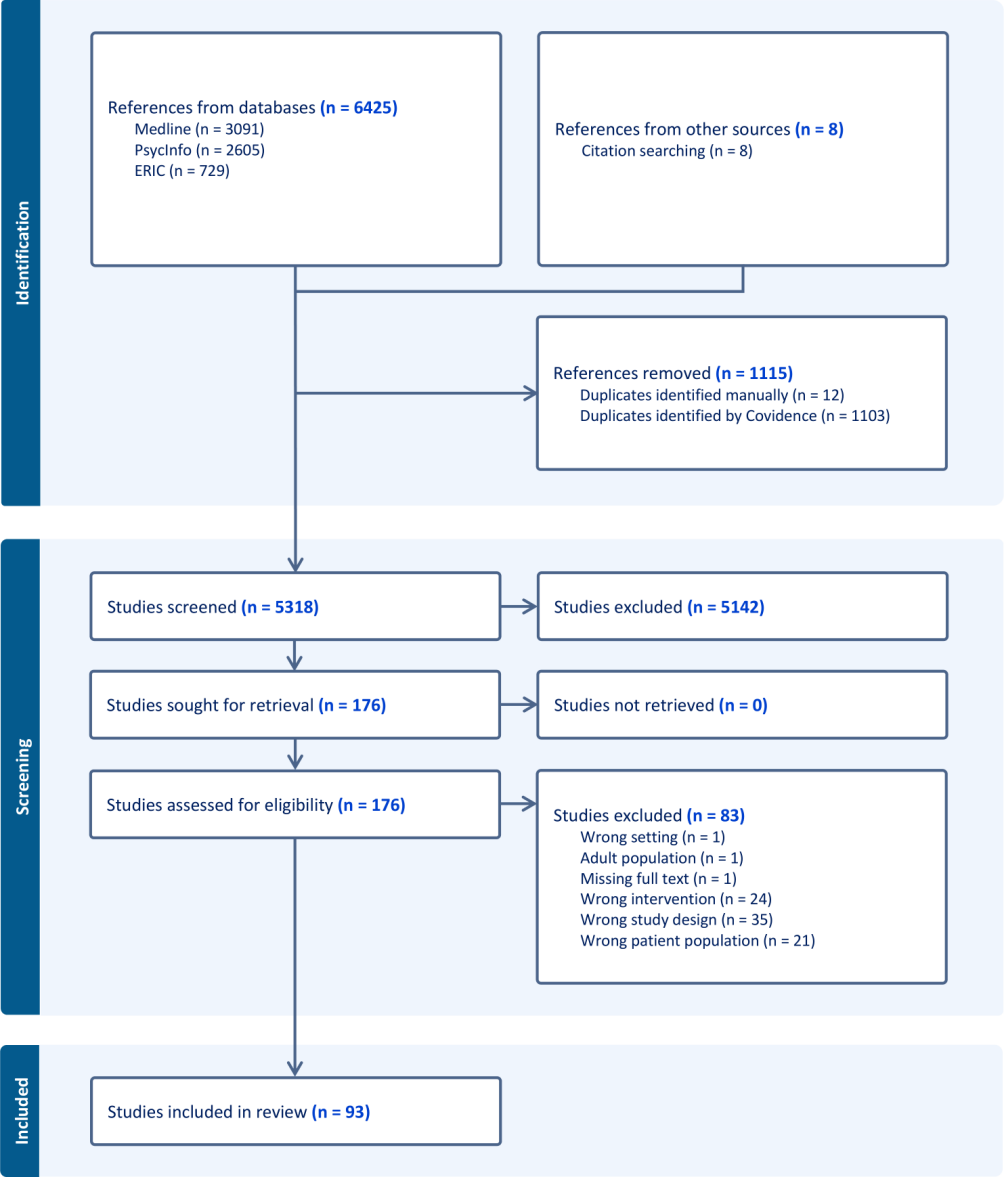
Preferred Reporting Items for Systematic reviews and Meta-Analyses extension for Scoping Reviews (PRISMA-ScR) evidence selection flow diagram.

Figure 1 presents the PRISMA flowchart. The search yielded 6433 articles, of which 5318 remained after duplicates were removed. Of those, 5142 were removed after title and abstract screening. The full texts of the remaining 176 articles were screened, and 83 were excluded due to various reasons (see figure 1), leaving 93 articles that met the inclusion criteria (see online supplemental material 2: Full reference list); the extracted data of the 93 articles can be found on the OSF depository of this review—https://osf.io/b68p3/?view_only=cc441a175cca4d7f85f5f6bba047808d.

### Characteristics of the included articles

**Figure 2:**
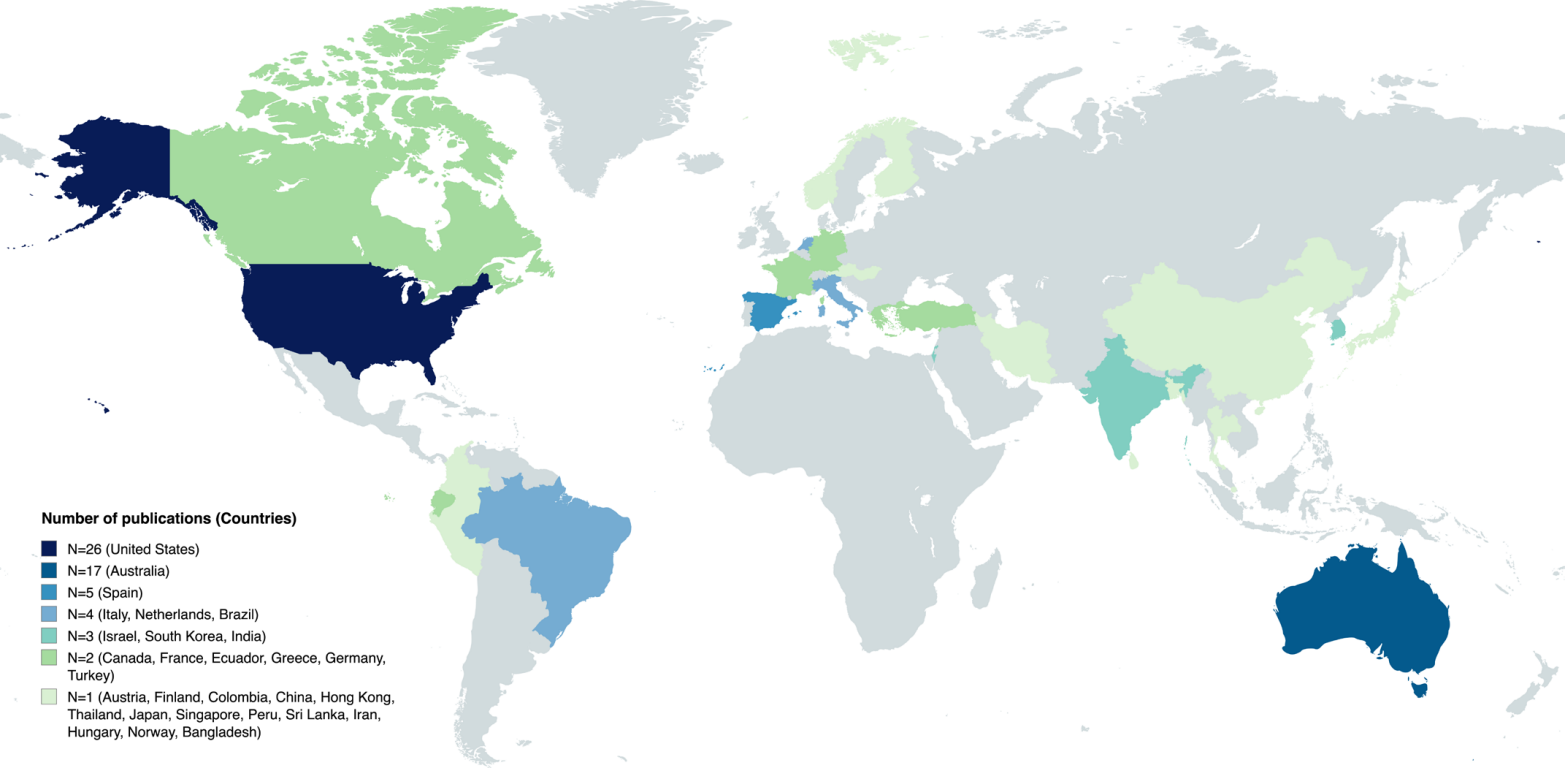
Global distribution of studies on healthy screen use interventions for adolescents.

This review includes 93 studies examining 81 distinct interventions to promote healthy screen use among adolescents. Eighty per cent (n=74) of these studies were conducted in high-income countries, with many in the USA (n=23, 25%) and Australia (n=18, 19%) (see figure 2). Fifteen per cent (n=14) were conducted in upper middle-income countries. Only 5% (n=5) were conducted in lower middle-income countries, with none conducted on the African continent. Seventy-eight per cent (n=73) examined interventions in school settings. Nine (10%) examined interventions that were web-based^22^ or application-based.^46^ Two studies (2%) focused on interventions in the home setting.^47 48^ Finally, nine (10%) studies focused on interventions in other settings such as driving setting,^46^ camps^49^ and mixed settings.^50^ Interventions were delivered by trained teachers, interventionists from the research teams and peers (eg, older students delivering interventions to younger ones).

In terms of study design, cluster-randomised controlled trials were the most common (n=43, 46%), followed by single-group pre-test and post-test studies (n=18, 19%), other quasi-experimental trials (n=14, 15%), post-test only studies (n=6, 6%), qualitative studies (ie, interviews/focus groups) (n=5, 5%), mixed method studies (n=3, 3%), prospective cohort studies (n=2, 2%) and pilot feasibility studies (n=2, 2%). Finally, 37% (n=34) of the studies were published within the past 5 years (2020 to 2024), and the remainder (n=59, 63%) were published between 2013 and 2019.

### Populations targeted

**Figure 3:**
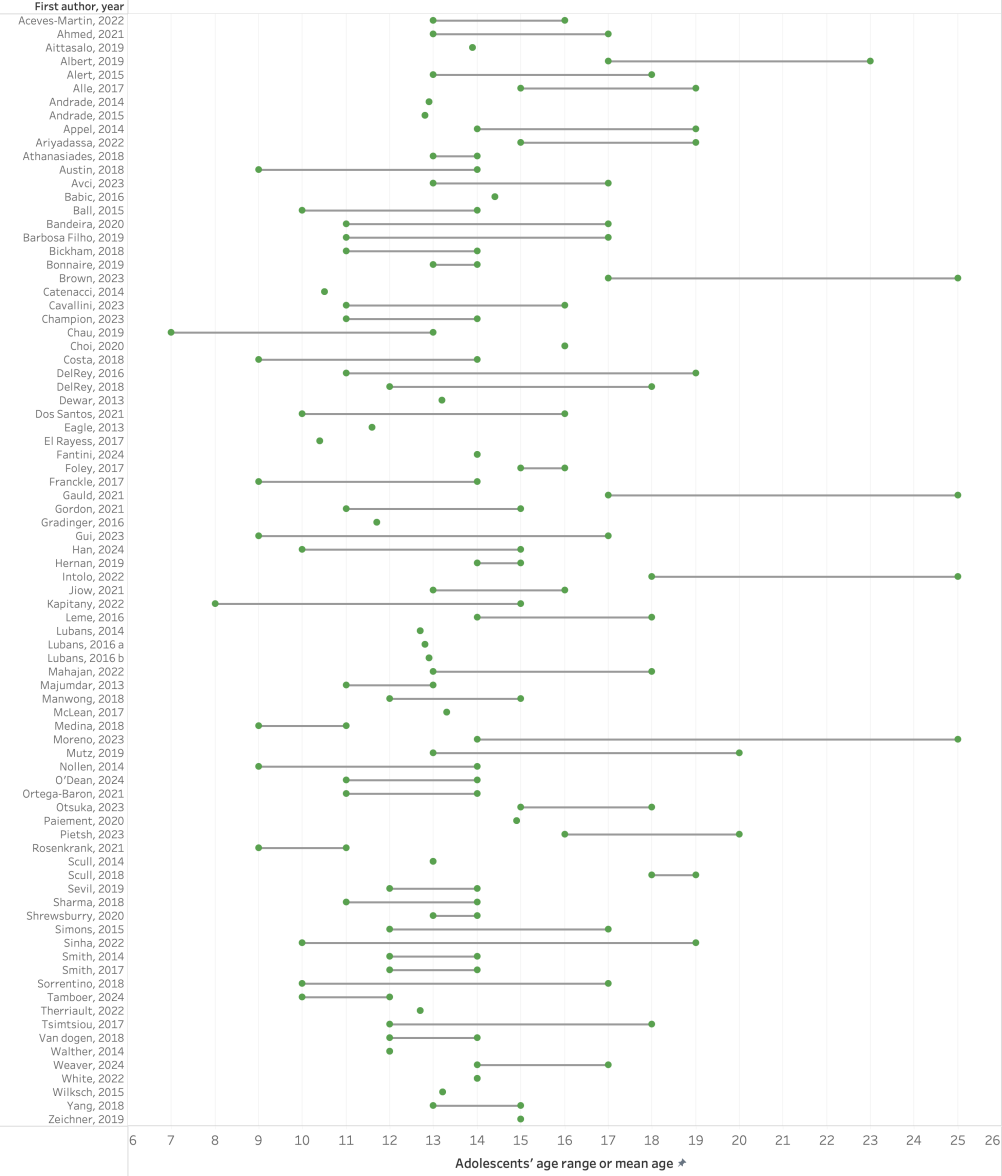
A description of adolescents’ age ranges or mean ages in the included studies. Bars represent age range, and dots represent mean age reported in the study. Articles that did not mention age (range or mean) are not represented.

Across the 93 included studies, an estimated total of 75 262 adolescents were represented, based on available sample size data reported by each study. Figure 3 shows that the age ranges of adolescents varied widely among the studies. A small number of studies included other types of participants than those for whom the intervention was directly intended, such as parents/guardians (three studies, n=1821), healthcare providers (two studies, n=295), teachers (one study, n=15) and peer leaders (one study, n=415).

### Theoretical and conceptual frameworks mobilised

Over a dozen theoretical frameworks or models were employed to underpin the studies or interventions. The combination of multiple theories within single study was also observed. Social cognitive theory was the most cited theory, used alone or in combination with other theories in 21 studies (23%).^19^ Thirteen studies (14%) used the self-determination theory.^51 52^ Other theoretical frameworks or models, such as the health action process approach,^53^ socioecological model,^20^ theory of planned behaviour,^54^ transtheoretical model,^55^ acceptance and commitment therapy,^56^ flow theory,^18^ cognitive dissonance theory^57^ and protection motivation theory,^58^ appeared less frequently. In terms of conceptual frameworks, the Health Promoting School Framework was the most frequently cited framework, used in seven studies (7.5%).^59 60^ Some less commonly adopted conceptual frameworks included Models of Decision-Making and Message Interpretation Process^61^ and the Healthy Internet Use model.^62^

### Evaluation approaches used

Outcome evaluation was the predominant approach used, with 75 articles (81%) focusing on assessing the direct effects (ie, effectiveness) of the interventions. Other methodologies were less frequently employed, including process evaluation (n=5, 5%) assessments of feasibility and acceptability (n=5, 5%) and comparisons of intervention modalities (n=7, 8%). One study used the Reach, Effectiveness, Adoption, Implementation, and Maintenance (RE-AIM) framework to examine the intervention’s reach, effectiveness, adoption, implementation and maintenance.^53^

### Functions of the included interventions

**Figure 4:**
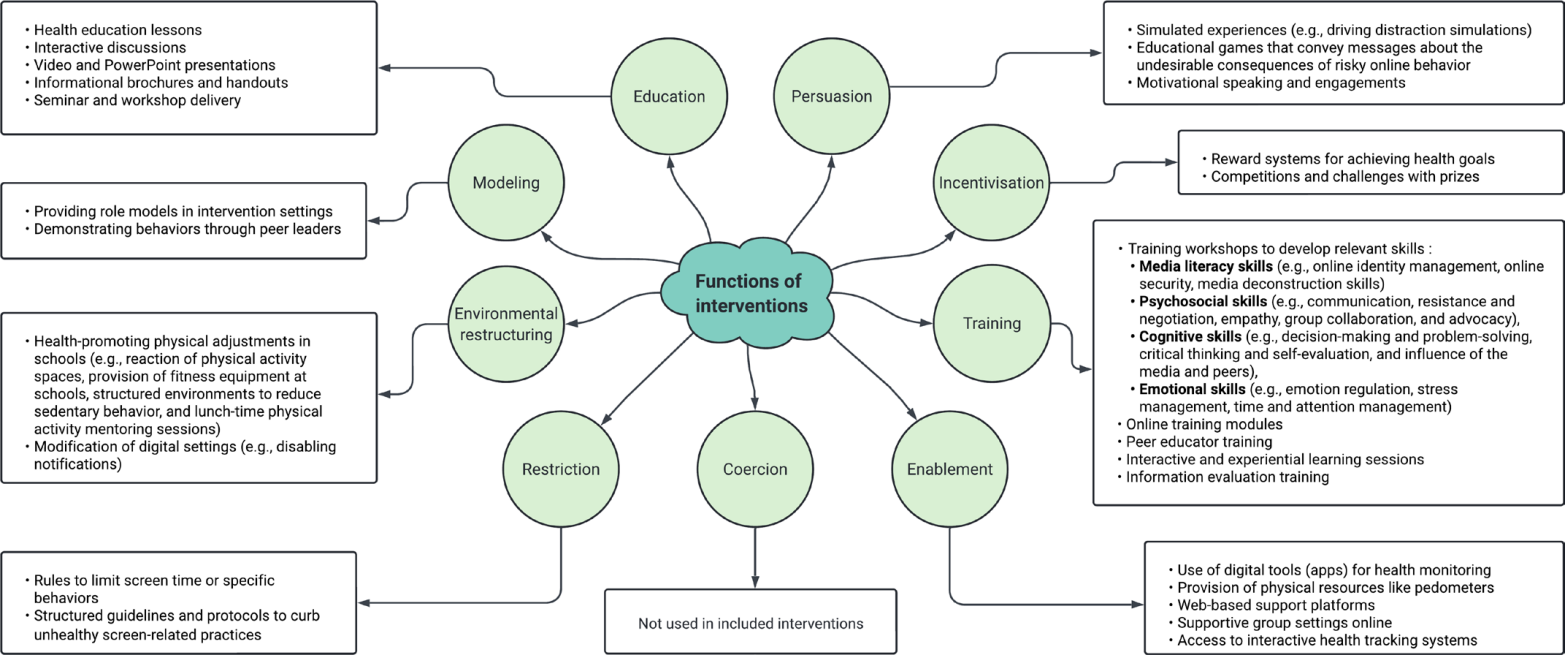
Intervention functions and corresponding strategies to promote healthy screen use among adolescents. Reference: Michie *et al*.^39^^(p7, table 1)^ The number and percentage of studies employing each function are provided in online supplemental material 3: functions of interventions used in included studies.

Figure 4 illustrates the intervention functions and examples of corresponding strategies used to promote healthy screen use behaviours among adolescents (see online supplemental material 3 for further details). Forty-five per cent of studies (n=42) incorporated two functions; 39% (n=36) included a single function; and 16% (n=15) involved three functions. Seventy-eight per cent (n=72) of the included studies examined interventions to educate adolescents, focused on raising awareness of risks associated with unhealthy screen use (eg, internet addiction) and on prevention strategies.^19^ Persuasive elements were present in interventions (n=4, 4%), such as that of Chau *et al*,^18^ which engaged participants in play-based activities that conveyed messages about the undesirable consequences of internet gaming disorder and risky online behaviour. Interventions involving incentivisation (n=2, 2%) and restriction (n=1, 1%), such as that of Hernan *et al*,^63^ managed screen use by implementing team-based rules (restricting cellphone use in class) and rewards (earning points for their teams). Training interventions were used in 34% of the studies (n=31). For example, Gui *et al*^50^ examined skills related to media literacy for teachers, time and attention management, communication and collaboration, and information evaluation. Enablement strategies (n=28, 30%) were used in an app-based intervention in which adolescents engaged in behaviour change challenges related to substance use, gambling and digital media use.^64^ None of the included studies used the coercion function. Some interventions targeted environmental restructuring (n=26, 28%), such as the ‘Active Teen Leaders Avoiding Screen-time’ trial, which integrated physical activity sessions and provided fitness equipment to reduce recreational screen time.^65^ Finally, modelling techniques (n=1, 1%) were employed in an internet addiction prevention intervention studied by Yang *et al*,^66^ which focused on role modelling (by peers and school nurses) and used case observations to provide vicarious experience aimed at enhancing self-regulatory efficacy.

### Duration of interventions and studies’ follow-up periods

The durations of the interventions varied. Some shorter interventions consisted of single one-time events, lasting from one half-hour^67^ (eg, a single educational presentation) to an entire day.^68^ Predominantly, the interventions studied were short term, lasting from a few sessions to several weeks (eg, one 90 min session per week for 5–10 weeks).^22 69^ Some more extensive school-based programmes lasted 4 to 6 months,^52 70^ or spanned up to 3 academic years.^71^ Follow-up periods for the studies examined varied significantly, ranging from immediate postintervention assessments^72^ to long-term evaluations extending up to 2 years.^73^ A majority of studies opted for short-term follow-ups to assess immediate effects of interventions (eg, pre and post-tests,^72^ 1-week,^74^ and 1-month follow-ups).^68^ Longitudinal follow-ups lasting two to 28 months^54^ were also observed.

### Outcomes targeted by the interventions

Twenty-five studies (27%) examined multiple health behaviours (eg, sedentary time,^53 75^ physical activity,^54 71^ including recreational screen time). Thirteen studies (14%) targeted screen time as the primary outcome.^52 70^ The remaining interventions targeted media literacy^76^ (n=10, 11%), cyberbullying^77^ (n=9, 10%), internet addiction^19^ (n=9, 10%), gaming addiction^78^ (n=6, 6%), safe internet use^79^ (n=5, 5%), social media use^80^ (n=4, 4%), screen use while driving^46^ (n=4, 4%), mental health^49^ (n=2, 2%) and sexual health^81^ (n=2, 2%).^81^ Single studies targeted posture, e-learning, password setting behaviour and mobile device use.

### Key findings

The studies demonstrated heterogeneous findings regarding the effectiveness of interventions to promote healthy screen use among adolescents. Although short-term improvements were frequently reported on various outcomes, such as reduced screen time and improved physical activity, long-term sustainability was less consistent across studies. Although 56 studies (60%) reported some positive intervention effects, about 20% (n=19) did not show statistically significant findings on screen-use related outcomes. For instance, the Health4Life intervention, a school-based educational e-Health programme, was tested among 6640 Australian adolescents 11–14 years old.^82 83^ The authors reported that Health4Life was not statistically more effective than usual school health education for influencing changes in any health behaviours, including screen time.

Interventions that targeted multiple health behaviours showed effectiveness in reorienting adolescents from screen time to alternative health behaviours, such as physical activity (n=15, 16%). For instance, the study of a school-based intervention tested among 320 adolescents aged 13–17 years indicated that the intervention increased physical activity levels and reduced sedentary and screen time among participants.^59^ Another study examining the effectiveness of Project Healthy Schools interventions among 2118 adolescents showed that the outcomes correlated with increases in physical activity and decreases in screen time.^84^

The review revealed a few promising interventions targeting screen-related behaviours that could be tested in other settings. In France, Bonnaire *et al* evaluated a 90 min prevention intervention that significantly changed adolescents’ perceptions of internet and gaming disorders while reducing time spent on the internet and video games.^78^ Similarly, the Social Competence Program in Austria, tested among 2042 students (mean age 11·7 years) in 18 schools, successfully prevented cyberbullying and cybervictimisation, with effects lasting 6 months, indicating sustainable benefits of systematic training in cyber safety and social skills.^20^ In the USA, Bickham and colleagues reported that the Take the Challenge programme, combining media education with a screen-free challenge, effectively reduced screen time (television viewing, background television time, after-school video gaming and weekend internet use) among participants, demonstrating the efficacy of combining educational content with environmental restructuring strategies.^85^ These interventions not only directly address behaviours related to screen use but also enhance the social and psychological competencies needed to navigate the digital world.

### Lessons learned from studying intervention implementation processes

Studies that examined feasibility, acceptability or implementation processes uncovered several key concerns and factors to be considered when designing interventions to promote healthy screen use among adolescents. The authors of a feasibility study of a school-based health and well-being programme that demonstrated high feasibility and implementation adherence recommended implementing such programmes as an elective course in secondary schools.^56^ Cavallini *et al* highlighted the importance of involving parents to reinforce behaviours promoted in school-based media education interventions.^86^ Another study, which tested a gaming and internet addiction prevention programme, highlighted the need for greater governmental involvement in promoting healthy use of internet and video games among adolescents.^78^

### Considerations for equity or differing effects based on population subgroups

The scoping review revealed heterogeneous effects of interventions aimed at promoting healthy screen use among adolescents, with differences notably associated with adolescents’ sex. Sex-specific interventions have been developed to address the unique needs and behaviours of different genders in relation to screen use and physical activity. For instance, the Nutrition and Enjoyable Activity for Teen Girls intervention is a 24-month school-based obesity prevention programme specifically designed for girls, focusing on nutrition and physical activity.^87 88^ On the other hand, Active Teen Leaders Avoiding Screentime is an obesity prevention programme targeting adolescent boys that uses a smartphone application to encourage physical activity and reduce screen time.^89^ Results indicated that certain interventions were more effective for boys. For instance, a cyberbullying prevention programme tested among 857 adolescents in Spain had stronger effects in heightening affective empathy among male participants.^74^ In contrast, some interventions demonstrated greater improvements in social media literacy,^90^ compulsive gaming and compulsive internet use among girls^71^ as compared with boys.^74^ Additionally, although less frequently addressed, some interventions specifically targeted adolescent populations who face socioeconomic and geographical barriers, including those from low-income communities (n=2, 2%),^65 91^ rural areas (n=2, 2%)^92 93^ and ethnic minority groups (n=1, 1%).^94^

## Discussion

This scoping review offers a comprehensive synthesis of empirical studies examining interventions that promote healthy screen use among adolescents. To our knowledge, this is the first effort to map comprehensively the existing literature on this subject using the intervention function framework.^39^ Using Michie *et al*’s framework, which outlines five intervention functions focusing on behaviour change (education, persuasion, incentivisation, training and enablement) and four functions addressing external influences on person agency (coercion, restriction, environmental restructuring and modelling),^39^ we observed that a majority of interventions used education and training functions while the four functions that emphasise external influences on agency were studied comparatively less. Although educational interventions are foundational, evidence shows that knowledge alone is insufficient to drive behaviour change.^95^ According to the ecological model of health promotion,^96^ educational interventions often operate only at the individual level while neglecting the broader social and environmental factors that shape screen use behaviours (physical environment and relational factors such as support from family, peers, school and community).^97^ In our review, several of the interventions that successfully reoriented adolescents away from excessive screen use towards alternative behaviours employed multiple intervention functions in parallel.^59^ These multicomponent strategies suggest that addressing the cognitive, behavioural and environmental drivers of screen use simultaneously may be more effective.

Furthermore, none of the interventions examined in these studies adopted coercion strategies, and only a few used restriction strategies to limit screen use at school or at home. Although some jurisdictions have introduced restrictive school phone policies or guidance (including England, France, Israel and Turkey, as well as regions of Canada and Australia),^98 99^ existing studies do not provide evidence for or against the benefits of restrictive school policies in promoting more healthful phone and social media use overall or better mental well-being in adolescents.^100^ In addition, the South Korean government implemented the ‘shutdown policy’ in 2011, which prohibited youths below age 16 years from playing online games between midnight and 06:00.^101^ However, the policy was eventually abolished in 2021 due to its limited effectiveness, concerns related to human rights and inappropriate regulation of the game industry.^101^ These findings suggest that the solution may not reside solely in restrictive strategies either. Findings of the scoping review also suggest that multilevel approaches could be further explored. In this review, the majority of interventions (78%) were conducted in school settings. This emphasis reveals a significant gap in incorporating home-based strategies that involve parents or parent–adolescent dyads. Furthermore, only a few interventions in this scoping review targeted key stakeholders in promoting healthy screen use in adolescents, such as parents and guardians. This gap is noteworthy, as familial involvement is important in shaping adolescents’ digital habits and mitigating risks associated with screen use.^102^ Parents often feel disempowered to regulate or counteract excessive screen use at home due to a lack of resources, tools or strategies that align with those taught in schools.^103^ This finding points to a critical need to develop interventions that support parents and guardians in establishing healthy screen use norms within the family environment. A preventive intervention for parents could, for instance, help increase their knowledge of video games and sense of personal effectiveness as parents by providing advice on how to monitor the use of video games (eg, playing times, use of a connection management system). Furthermore, this scoping review underscores the potential benefits of adopting the Medical Research Council’s framework for developing and evaluating complex interventions.^104^ This framework emphasises the importance of context, interactions between interventions and their settings and the inclusion of diverse stakeholder perspectives, which are crucial for developing effective and adaptable strategies for promoting healthy screen use.

Few studies in this scoping review explicitly addressed equity, such as how interventions’ effectiveness might differ across socioeconomic or cultural groups. Yet research consistently shows that digital inequalities (eg, unequal access to resources) influence screen use behaviours and associated risks.^105^ Moreover, a majority of studies (80%) were conducted in high-income countries, particularly the USA and Australia, with limited representation from low and middle-income countries. This geographic bias reflects broader trends in public health research, where low and middle-income countries are under-represented, despite significant shifts in screen use behaviours in these regions due to increased prevalence of smartphone and digital devices.^106^ Interventions developed in high-income countries may not translate well to low and middle-income countries, where family structures, school resources and societal norms regarding screen use differ significantly.^107^ Furthermore, cultural differences in screen use may require culturally adapted interventions. For instance, a systematic review and meta-analysis on the prevalence of social media addiction across 32 nations revealed that social media addiction is more prevalent in collectivist countries (31%) than in individualist countries (14%).^108^ That is, echoing work by Hofstede,^109^ Cheng *et al* state that collectivist cultures often emphasise tight-knit community and family ties, which can increase pressure on individuals to engage online to maintain these connections. In contrast, individualist cultures prioritise personal independence, with potentially less pressure to maintain constant social media engagement.^108^ This disparity may stem from cultural differences in how individuals interact within their social networks.^108^ Including these cultural considerations might enhance the effectiveness of future interventions targeting adolescents from different cultural backgrounds.

Furthermore, although a few of the interventions were sex-specific, none specifically addressed gender identity. However, recent research reported that adolescents who identify with a sexual minority group have higher problematic social media, video game and mobile phone use, compared with heterosexual adolescents.^110^ Other studies showed that sexual minority adolescents face increased risk of cybervictimisation.^111^ These findings point to a need to develop evidence‐based prevention programmes that effectively address the complexities of minoritised identities and healthy screen use in adolescents. In sum, future interventions could incorporate equity-focused design and evaluation to ensure they respond to the needs of diverse populations.

We also observed a wide array of outcomes targeted by interventions aimed at promoting healthy screen use among adolescents. These included reducing overall screen time, enhancing media literacy, preventing cyberbullying and fostering safe internet use practices. Although these outcomes are critical for cultivating healthy screen use, certain other outcomes, such as the prevention of gaming addiction, received relatively less attention. Only a limited number of interventions (n=6) specifically addressed gaming addiction prevention among adolescents. Even though gaming has been associated with potential benefits such as improved visual-spatial skills, enhanced problem-solving abilities and increased prosocial interaction,^112^ it has also been increasingly recognised for its potential to contribute to behavioural issues such as aggression, cyberbullying and verbal abuse.^113^ The limited focus on healthy gaming behaviours may reflect unequal emphasis on screen time quantity over content or contextual factors. As digital landscapes evolve, interventions must adapt to address not only how much adolescents engage with screens but also how they engage, particularly in environments eliciting addiction. These considerations are consistent with Ferrari and Schick’s view that the impact of screen use among adolescents is shaped not only by duration but also by content, context and individual-level factors, all of which warrant closer attention in both research and intervention design.^114^

Finally, we propose 10 future directions for intervention and research, summarised in table 2. These recommendations aim to address current gaps and enhance the positive impact and applicability of future initiatives in promoting healthy screen use among adolescents.

**Table 2:** Proposed future directions for intervention and research

Future direction	Details
Engage stakeholders	Increasing engagement with stakeholders, including educators, parents, health professionals, policymakers and especially adolescents, in designing and implementing interventions (ie, using participatory and co-designed approaches). This could enhance interventions’ relevance and applicability and facilitate their integration into existing systems.
Explore intervention functions	Exploring intervention functions that emphasise external influences (ie, modelling, environmental restructuring, restriction, coercion), as these have been studied less compared with other intervention functions (eg, education, training).
Target marginalised groups	Targeting marginalised, underrepresented and/or sexual minority populations to ensure interventions are equitable and effective across different groups.
Develop comprehensive interventions	Developing and implementing interventions that address all major dimensions of healthy screen use to support adolescent well-being comprehensively, without neglecting important outcomes (eg, prevention of video game addiction).
Address developmental differences	Addressing developmental differences in their target population/age groups (eg, cognitive maturity, digital literacy).
Clarify theories of change	Including more explicit depictions and narratives of underlying theories of change.
Define ‘healthy screen use’	Clarifying the definition and measurement of ‘healthy screen use’ to identify key intervention goals and strengthen comparability of investigations.
Use diverse research designs	Applying a greater diversity of experimental and quasi-experimental designs to ascertain adequately the overall impact of interventions as well as heterogeneity across contexts and individual characteristics.
Examine implementation processes	Examining interventions’ implementation processes, including facilitators and barriers to successful delivery, participant engagement and contextual factors influencing intervention outcomes.
Conduct meta-analyses in systematic reviews	Conducting meta-analyses to synthesise findings from multiple studies and to quantify the overall effect sizes of interventions while examining short-term and long-term intervention effects to address sustainability over time.

### Limitations

This scoping review has several limitations. First, our review did not include grey literature, such as governmental or organisational reports, which might contain evidence relevant to policies implemented to promote healthy screen use among adolescents. Second, we also excluded non-empirical studies, such as discussion papers on intervention development or methodological papers, which could provide insights into intervention design and implementation processes. Finally, it should be noted that this review only focused on interventions. Future research should attempt to synthesise evidence on policies aimed at promoting healthy screen use among adolescents.

### Conclusions

The findings of this scoping review reveal that interventions implemented to promote healthy screen use vary extensively, on diverse conceptual and methodological aspects, but most notably in their effectiveness. This scoping review indicates a substantial gap in our capability to foster healthier screen use behaviour systematically and equitably among adolescents. This underscores the urgent need to enhance and refine intervention strategies and evaluate their effects over time. We call for a concerted effort among researchers, policymakers and practitioners to establish a clear agenda prioritising the development and evaluation of interventions that promote healthy screen use behaviours equitably among adolescents.

## Supplementary material

10.1136/bmjopen-2025-103772 online supplemental file 1

## Data Availability

All data supporting the results reported in this review can be found on the Open Science Framework (OSF) platform (https://doi.org/10.17605/OSF.IO/B68P3).
