# Supplementary material for: Scoping review of interventions aimed at promoting healthy screen use among adolescents
Source: BMJ Open. 2025 Sep 21;15(9):e103772. doi: 10.1136/bmjopen-2025-103772 (PMC12458889; doi:10.1136/bmjopen-2025-103772)
Supplement: online supplemental file 1 [file bmjopen-15-9-s001.pdf]

**Manuscript title: A Scoping Review of Interventions Aimed at Promoting Healthy Screen Use Among Adolescents**

## Supplementary materials table of contents


| | Page numbers |
|---|---|
| Supplementary material 1. Full search strategies | **1 – 7** |
| Supplementary material 2. Full reference list of the 93 included articles | **8 – 13** |
| Supplementary material 3. Function of intervention used in the included studies | **14 – 16** |

## Supplementary material 1. Full search strategies

**05/06/2024**

**Ovid MEDLINE(R) ALL <1946 to June 04, 2024>**

| | | |
|---|---|---|
| 1 | (teen* or youth* or adolescen* or (young adj2 (person* or individual*)) or highschool* or ((secondary or high*) adj2 (school* or education))).ab,ti,kf. | 542696 |
| 2 | adolescent/ | 2251565 |
| 3 | 1 or 2 | 2425157 |
| 4 | ("screen time" or screentime or ((screen or screens) adj3 (usage or "use")) or "Screen-based sedentary behavior" or (("Video game*" or "Video gaming*" or "social media" or Facebook or YouTube or Twitter or Snapchat or Instagram or TikTok or "WhatsApp*" or "mobile phone*" or "smart phone*" or "smartphone*" or "mobile device*" or "digital media" or "cell phone*" or "cellphone*" or "cellular phone*" or "mobile app*" or internet) adj3 ("use" or usage or addiction* or problemat* or literac*))).ab,ti,kf. | 32062 |
| 5 | Screen time/ or technology addiction/ or internet addiction disorder/ or "Internet use"/ | 3001 |
| 6 | (Smartphone/ or cell phone/ or social media/) and "Behavior, Addictive"/ | 695 |
| 7 | 4 or 5 or 6 | 32700 |
| 8 | (intervention* or program* or strateg* or "health education" or family-based or community-based or School-based).ab,ti,kf. | 3779859 |
| 9 | Health Education/ | 64297 |
| 10 | 8 or 9 | 3810814 |
| 11 | 3 and 7 and 10 | 4016 |
| 12 | Case Reports/ | 2405525 |
| 13 | Organizational Case Studies/ | 12652 |
| 14 | Qualitative Research/ | 88485 |
| 15 | qualitative research*.mp. | 109539 |
| 16 | qualitative stud*.mp. | 75543 |
| 17 | action research.mp. | 5976 |
| 18 | Community-Based Participatory Research/ | 5867 |
| 19 | participatory research.mp. | 9753 |
| 20 | case stud*.mp. | 146135 |
| 21 | ethno*.mp. | 211499 |
| 22 | grounded theory.mp. | 16028 |
| 23 | phenomeno*.mp. | 294732 |
| 24 | Narration/ | 10582 |

| | | |
|---|---|---|
| 25 | narrative*.mp. | 92703 |
| 26 | biograph*.mp. | 187624 |
| 27 | Autobiography/ | 3967 |
| 28 | Autobiograph*.mp. | 10660 |
| 29 | documentar*.mp. | 3853 |
| 30 | qualitative synthes*.mp. | 3621 |
| 31 | active feedback.mp. | 308 |
| 32 | conversation*.mp. | 30228 |
| 33 | discourse*.mp. | 22088 |
| 34 | thematic.mp. | 68474 |
| 35 | qualitative data.mp. | 26374 |
| 36 | key informant*.mp. | 11778 |
| 37 | Focus Groups/ | 37421 |
| 38 | focus group*.mp. | 73821 |
| 39 | case report*.mp. | 2503579 |
| 40 | Interview/ | 31035 |
| 41 | interview*.mp. | 511025 |
| 42 | Observation/ | 5919 |
| 43 | observer*.mp. | 111496 |
| 44 | visual data.mp. | 862 |
| 45 | (audio adj record*).mp. | 11974 |
| 46 | Anthropology, Cultural/ | 7728 |
| 47 | experience*.mp. | 1422732 |
| 48 | or/12-47 | 5141558 |
| 49 | exp clinical trial/ | 995847 |
| 50 | exp Research Design/ | 502764 |
| 51 | random allocation/ | 107282 |
| 52 | double-blind method/ | 178810 |
| 53 | Single-Blind Method/ | 33551 |
| 54 | Placebos/ | 35965 |
| 55 | Cross-Over Studies/ | 56790 |
| 56 | or/49-55 | 1329938 |
| 57 | (clinic* adj25 trial*).mp. | 1309085 |

| | | |
|---|---|---|
| 58 | random*.mp. | 1751166 |
| 59 | control*.mp. | 6540568 |
| 60 | (latin adj square).mp. | 5555 |
| 61 | placebo*.mp. | 269112 |
| 62 | or/57-61 | 7634489 |
| 63 | Comparative Study/ | 1918043 |
| 64 | comparative stud*.mp. | 1990107 |
| 65 | Validation Studies/ | 110244 |
| 66 | validation stud*.mp. | 130481 |
| 67 | evaluation studies/ | 262489 |
| 68 | evaluation stud*.mp. | 391244 |
| 69 | Follow-Up Studies/ | 698588 |
| 70 | followup.mp. | 21315 |
| 71 | follow-up.mp. | 1635559 |
| 72 | Prospective Studies/ | 689279 |
| 73 | Cross-Over Studies/ | 56790 |
| 74 | cross over.mp. | 72059 |
| 75 | crossover.mp. | 75918 |
| 76 | prospective*.mp. | 1096993 |
| 77 | volunteer*.mp. | 244814 |
| 78 | or/63-77 | 4783636 |
| 79 | singl*.mp. | 2262415 |
| 80 | doubl*.mp. | 753452 |
| 81 | trebl*.mp. | 553 |
| 82 | tripl*.mp. | 170941 |
| 83 | or/79-82 | 3001002 |
| 84 | mask*.mp. | 113537 |
| 85 | blind*.mp. | 433738 |
| 86 | 84 or 85 | 539121 |
| 87 | 83 and 86 | 304366 |
| 88 | 56 or 62 or 78 or 87 | 10906656 |
| 89 | Cohort Studies/ | 342262 |
| 90 | Case-Control Studies/ | 334864 |

| | | |
|---|---|---|
| 91 | Cross-Sectional Studies/ | 504282 |
| 92 | Health Surveys/ | 67544 |
| 93 | Health Care Surveys/ | 34128 |
| 94 | Risk/ | 128842 |
| 95 | Incidence/ | 307594 |
| 96 | Prevalence/ | 353583 |
| 97 | Mortality/ | 49989 |
| 98 | cohort*.mp. | 1036615 |
| 99 | case-control.mp. | 390961 |
| 100 | cross sectional.mp. | 697503 |
| 101 | (health* adj2 survey*).mp. | 154126 |
| 102 | risk.mp. | 3367942 |
| 103 | incidence.mp. | 1063948 |
| 104 | prevalence.mp. | 923797 |
| 105 | mortality.tw. | 1020704 |
| 106 | case series.mp. | 110010 |
| 107 | time series.mp. | 49801 |
| 108 | (before and after).mp. [mp=title, book title, abstract, original title, name of substance word, subject heading word, floating sub-heading word, keyword heading word, organism supplementary concept word, protocol supplementary concept word, rare disease supplementary concept word, unique identifier, synonyms, population supplementary concept word, anatomy supplementary concept word] | 915007 |
| 109 | prognos*.mp. | 1137245 |
| 110 | predict*.mp. | 2256942 |
| 111 | course*.mp. | 725830 |
| 112 | or/89-111 | 9080916 |
| 113 | (mixed adj5 method*).mp. | 55270 |
| 114 | multimethod*.mp. | 2678 |
| 115 | (multiple adj5 method*).mp. | 38380 |
| 116 | or/113-115 | 95590 |
| 117 | qualitative.mp. | 366554 |
| 118 | Qualitative Research/ | 88485 |
| 119 | quantitative.mp. | 821059 |

| | | |
|---|---|---|
| 120 | 117 or 118 | 366554 |
| 121 | 119 and 120 | 109738 |
| 122 | 116 or 121 | 189615 |
| 123 | 48 or 88 or 112 or 122 | 18507842 |
| 124 | 123 not (letter or comment or editorial or newspaper article).pt. | 17748551 |
| 125 | 124 not (exp animals/ not humans.sh.) | 15689794 |
| 126 | 11 and 125 | 3597 |
| 127 | limit 126 to yr="2013 -Current" | 3091 |

| | **ERIC <1965 to April 2024>** | **05/06/2024** |
|---|---|---|
| 1 | (teen* or youth* or adolescen* or (young adj2 (person* or individual*)) or highschool* or ((secondary or high*) adj2 (school* or education))).ab,ti,id. | 361510 |
| 2 | Adolescents/ | 55478 |
| 3 | 1 or 2 | 372091 |
| 4 | ("screen time" or screentime or ((screen or screens) adj3 (usage or "use")) or "Screen-based sedentary behavior" or (("Video game*" or "Video gaming*" or "social media" or Facebook or YouTube or Twitter or Snapchat or Instagram or TikTok or "WhatsApp*" or "mobile phone*" or "smart phone*" or "smartphone*" or "mobile device*" or "digital media" or "cell phone*" or "cellphone*" or "cellular phone*" or "mobile app*" or internet) adj3 ("use" or usage or addiction* or problemat* or literac*))).ab,ti,id. | 7194 |
| 5 | media literacy/ or "computer use "/ or Digital Literacy/ | 4002 |
| 6 | (Social media/ or handheld devices/ or computer games/ or internet/) and Addictive Behavior/ | 583 |
| 7 | 4 or 5 or 6 | 10730 |
| 8 | (intervention* or program* or strateg* or "health education" or family-based or community-based or School-based).ab,ti,id. | 671080 |
| 9 | exp intervention/ or exp programs/ or exp health education/ or health programs/ | 312454 |
| 10 | 8 or 9 | 768852 |
| 11 | 3 and 7 and 10 | 1069 |
| 12 | Social media/ or media literacy/ or "computer use"/ or Digital Literacy/ | 9442 |
| 13 | 4 or 12 | 14521 |
| 14 | 3 and 10 and 13 | 1424 |
| 15 | 14 not 11 | 366 |
| 16 | limit 11 to yr="2013 -Current" | 729 |

| | **APA PsycInfo <1806 to May Week 5 2024>** | **05/06/2024** |
|---|---|---|
| 1 | (teen* or youth* or adolescen* or (young adj2 (person* or individual*)) or highschool* or ((secondary or high*) adj2 (school* or education))).ab,ti,id. | 517776 |
| 2 | 200.ag. | 519265 |
| 3 | 1 or 2 | 746896 |
| 4 | ("screen time" or screentime or ((screen or screens) adj3 (usage or "use")) or "Screen-based sedentary behavior" or (("Video game*" or "Video gaming*" or "social media" or Facebook or YouTube or Twitter or Snapchat or Instagram or TikTok or "WhatsApp*" or "mobile phone*" or "smart phone*" or "smartphone*" or "mobile device*" or "digital media" or "cell phone*" or "cellphone*" or "cellular phone*" or "mobile app*" or internet) adj3 ("use" or usage or addiction* or problemat* or literac*))).ab,ti,id. | 25396 |
| 5 | screen time/ or computer usage/ or internet addiction/ or internet usage/ or "smartphone use"/ or Digital Literacy/ | 11472 |
| 6 | (exp social media/ or internet/ or exp mobile phones/ or mobile applications/) and exp addiction/ | 865 |
| 7 | 4 or 5 or 6 | 29512 |
| 8 | (intervention* or program* or strateg* or "health education" or family-based or community-based or School-based).ab,ti,id. | 1207734 |
| 9 | School Based Intervention/ or Intervention/ or health education/ or public health campaigns/ | 130746 |
| 10 | 8 or 9 | 1214646 |
| 11 | 3 and 7 and 10 | 3067 |
| 12 | limit 11 to yr="2013 -Current" | 2605 |

**Supplementary material 2. Full reference list of the 93 included studies**


1. Aceves-Martins M, Llauradó E, Tarro L, et al. "Som la Pera," a school-based, peer-led social marketing intervention to engage spanish adolescents in a healthy lifestyle: a parallel-cluster randomized controlled study. *Child Obes* 2022;18(8):556-571. doi:10.1089/chi.2021.0207
2. Ahmed KR, Kolbe-Alexander T, Khan A. Effectiveness of a school-based intervention on physical activity and screen time among adolescents. *J Sci Med Sport* 2022; 25(3): 242–8. doi:10.1016/j.jsams.2021.10.007
3. Aittasalo M, Jussila A-M, Tokola K, Sievänen H, Vähä-Ypyä H, Vasankari T. Kids Out; evaluation of a brief multimodal cluster randomized intervention integrated in health education lessons to increase physical activity and reduce sedentary behavior among eighth graders. *BMC Public Health* 2019; 19(1): 415. doi:10.1186/s12889-019-6737-x
4. Aizenkot D. Cyberbullying experiences in classmates' WhatsApp discourse, across public and private contexts. *Child Youth Serv Rev* 2020; 110: 104814. doi:10.1016/j.childyouth.2020.104814
5. Albert G, Lotan T. Exploring the impact of "soft blocking" on smartphone usage of young drivers. *Accid Anal Prev* 2019; 125: 56–62. doi:10.1016/j.aap.2019.01.031
6. Alert MD, Carucci D, Clennan MK, Chiles S, Etzel EN, Saab PG. Reducing obesity in students everywhere (rose): a brief, interactive, school-based approach to promoting health. *Journal of Health Education Teaching* 2015; 6(1):72-86.
7. Allee L, Dechert T, Rao SR, et al. The eastern association for the surgery of trauma's injury control and violence prevention committee's annual distracted driving outreach event: Evaluating attitude and behavior change in high school students. *Journal of trauma and acute care surgery* 2018;84(1):31-36. doi:10.1097/TA.0000000000001589
8. Andrade S, Lachat C, Ochoa-Aviles A, et al. A school-based intervention improves physical fitness in Ecuadorian adolescents: a cluster-randomized controlled trial. *Int J Behav Nutr Phys Act* 2014; 11(1): 153. doi:10.1186/s12966-014-0153-5
9. Andrade S, Verloigne M, Cardon G, et al. School-based intervention on healthy behaviour among Ecuadorian adolescents: effect of a cluster-randomized controlled trial on screen-time. *BMC public health* 2015;15:942. doi:10.1186/s12889-015-2274-4
10. Appel HB, Huang B, Cole A, James R, Ai AL. Starting the conversation - a childhood obesity knowledge project using an app. Br J Med Med Res 2014; 4(7): 1526–38. doi:10.9734/bjmmr/2014/5512
11. Ariyadasa G, De Silva C, Gamagedara NS. Educational intervention for the prevention of internet addiction disorder among 15-19-year-old adolescents in Colombo district, Sri Lanka. Ceylon Med J 2022; 67(4): 131–7. doi:10.4038/cmj.v67i4.9740
12. Athanasiades C, Kamariotis T, Psalti A, Baldry A, Sorrentino A. Internet use and cyberbullying among adolescent students in Greece: the tabby project. *Hellenic Journal of Psychology* 2015;12:14-39.
13. Austin EW, Austin BW, French BF, Cohen MA. The effects of a nutrition media literacy intervention on parents' and youths' communication about food. *J Health Commun* 2018; 23(2): 190–9. doi:10.1080/10810730.2018.1423649
14. Avci D, Gündoğdu NA, Dönmez RH, Avci FE. Students as teachers: effect of the peer education model on reducing smartphone addiction in adolescents. *Health Education Research* 2023;38(2):107-118. doi:10.1093/her/cyac042
15. Babic MJ, Smith JJ, Morgan PJ, et al. Intervention to reduce recreational screen-time in adolescents: outcomes and mediators from the 'Switch-Off 4 Healthy Minds' (S4HM) cluster randomized controlled trial. *Prev Med* 2016; 91: 50–7. doi:10.1016/j.ypmed.2016.07.014
16. Pal Singh Balhara Y, Singh S. Online course on basics of management of behavioral addictions involving use of internet: observations from the first batch of participants. *Asian J Psychiatr* 2019; 44: 1–3. doi:10.1016/j.ajp.2019.07.013
17. Ball SD, Kovarik J, Leidy HJ. Active and healthy schools. *The Physical Educator* 2015;72
18. Bandeira ADS, Silva KS, Bastos JLD, Silva DAS, Lopes ADS, Barbosa Filho VC. Psychosocial mediators of screen time reduction after an intervention for students from schools in vulnerable areas: a cluster-randomized controlled trial. *J Sci Med Sport* 2020; 23(3): 264–9. doi:10.1016/j.jsams.2019.09.004

19. Barbosa Filho VC, Bandeira ADS, Minatto G, et al. Effect of a multicomponent intervention on lifestyle factors among Brazilian adolescents from low Human Development Index areas: a cluster-randomized controlled trial. *Int J Environ Res Public Health* 2019; 16(2): 267. doi:10.3390/ijerph16020267
20. Bickham DS, Hswen Y, Slaby RG, Rich M. A preliminary evaluation of a school-based media education and reduction intervention. J Prim Prev 2018; 39(3): 229–45. doi:10.1007/s10935-018-0510-2
21. Bonnaire C, Serehen Z, Phan O. Effects of a prevention intervention concerning screens, and video games in middle-school students: influences on beliefs and use. *J Behav Addict* 2019; 8(3): 537–53. doi:10.1556/2006.8.2019.54
22. Boor Boor B, Khodabakhshi-Koolaee A, Falsafinejad MR. The effect of the parent-child relationship enrichment training package with an emphasis on the internet use: a mixed methods approach. *Practice in Clinical Psychology* 2021;9(3):199-210. doi:10.32598/jpcp.9.3.746.2
23. Brown PM, George AM, van Boxtel JJA, Lewis A. Drive in the Moment: an evaluation of a web-based tool designed to reduce smartphone use among young drivers. *Traffic Inj Prev* 2023; 24(6): 466–74. doi:10.1080/15389588.2023.2218509
24. Busch V, De Leeuw JRJ, Zuithoff NP, Van Yperen TA, Schrijvers AJ. A controlled Health Promoting School study in the Netherlands: effects after 1 and 2 years of intervention. *Health Promot Pract* 2015; 16(4): 592–600. doi:10.1177/1524839914566272
25. Busch V, De Leeuw RJJ, Schrijvers AJP. Results of a multibehavioral health-promoting school pilot intervention in a Dutch secondary school. *J Adolesc Health* 2013; 52(4): 400–6. doi:10.1016/j.jadohealth.2012.07.008
26. Catenacci V, Barrett C, Odgen L, et al. Changes in physical activity and sedentary behavior in a randomized trial of an internet-based versus workbook-based family intervention study. *Journal of Physical Activity and Health* 2014;11(2):348-358. doi:10.1123/jpah.2012-0043
27. Cavallini MC, Caravita SCS, Fandrem H. Feasibility and early outcomes of a parent training intervention to engage parents in children's media education. *Healthcare (Basel)* 2023; 11(15); 2130. doi:10.3390/healthcare11152130
28. Celik C. Educational intervention for reducing internet addiction tendencies. *Addicta: The Turkish Journal on Addictions* 2017;3 doi:10.15805/addicta.2016.3.0021
29. Champion KE, Newton NC, Gardner LA, et al. Health4Life eHealth intervention to modify multiple lifestyle risk behaviours among adolescent students in Australia: a cluster-randomised controlled trial. *Lancet Digit Health* 2023; **5**(5): e276–87. doi:10.1016/s2589-7500(23)00028-6
30. Chau C-L, Tsui YY-Y, Cheng C. Gamification for internet gaming disorder prevention: evaluation of a wise it-use (wit) program for Hong Kong primary students. *Frontiers in Psychology* 2019;10 doi:10.3389/fpsyg.2019.02468
31. Choi EH, Chun MY, Lee I, Yoo YG, Kim MJ. The Effect of mind subtraction meditation intervention on smartphone addiction and the psychological wellbeing among adolescents. *Int J Environ Res Public Health* 2020;17(9)doi:10.3390/ijerph17093263
32. Costa C, Tyner K, Henriques S, Sousa C. Game creation in youth media and information literacy education. *Int J Game Based Learn* 2018; **8**(2): 1–13. doi:10.4018/IJGBL.2018040101
33. Cuesta Medina L, Hennig Manzuoli C, Duque LA, Malfasi S. Cyberbullying: tackling the silent enemy. *International Journal of Inclusive Education* 2020;24(9):936-947. doi:10.1080/13603116.2018.1500648
34. Del Rey R, Casas JA, Ortega R. Impact of the ConRed program on different cyberbulling roles. *Aggress Behav* 2016; **42**(2): 123–35. doi:10.1002/ab.21608
35. Del-Rey R, Mora-Merchán JA, Casas JA, Ortega-Ruiz R, Elipe P. 'Asegúrate' Program: Effects on cyber-aggression and its risk factors. *Comunicar*. 2018; 56(26): 39-48
36. Dewar DL, Morgan PJ, Plotnikoff RC, et al. The nutrition and enjoyable activity for teen girls study: a cluster randomized controlled trial. *Am J Prev Med* 2013; **45**(3): 313–7. doi:10.1016/j.amepre.2013.04.014
37. Dos Santos PC, Salmon J, Arundell L, Lopes MVV, Silva KS. Effectiveness and moderators of a multicomponent school-based intervention on screen time devices: the movimente cluster-randomized controlled trial. *BMC Public Health* 2021;21(1):1852. doi:10.1186/s12889-021-11895-2

38. Draper M, Appregilio S, Kramer A, et al. Educational intervention/case study: implementing an elementary-level, classroom-based media literacy education program for academically at-risk middle-school students in the non-classroom setting. *J Alcohol Drug Educ* 2015; **59**(2): 12–24.
39. Eagle TF, Gurm R, Smith CA, et al. A middle school intervention to improve health behaviors and reduce cardiac risk factors. *The American Journal of Medicine* 2013;126(10):903-908. doi:10.1016/j.amjmed.2013.04.019
40. El Rayess F, Gandhi M, Mennillo H. Mark, Set, Go! School-based nutrition and physical activity program: a five-year evaluation. *R I Med J* 2017;100(2):39-44.
41. Fantini L, Gostoli S, Artin MG, Rafanelli C. An intervention based on well-being therapy to prevent alcohol use and other unhealthy lifestyle behaviors among students: a three-arm cluster randomized controlled trial. *Psychol Health Med* 2024;29(5):930-950. doi:10.1080/13548506.2023.2235740
42. Foley BC, Shrewsbury VA, Hardy LL, Flood VM, Byth K, Shah S. Evaluation of a peer education program on student leaders' energy balance-related behaviors. *BMC Public Health* 2017;17(1):695. doi:10.1186/s12889-017-4707-8
43. Franckle RL, Falbe J, Gortmaker S, et al. Student obesity prevalence and behavioral outcomes for the massachusetts childhood obesity research demonstration project. *Obesity (Silver Spring)* 2017;25(7):1175-1182. doi:10.1002/oby.21867
44. Gauld C, Watson B, Lewis I, White KM, Pammer K. An exploration of the effectiveness of in-person and online versions of the induced hypocrisy paradigm to reduce smartphone use among young drivers. *Transp Res Part F Traffic Psychol Behav* 2021; **82**: 462–74. doi:10.1016/j.trf.2021.09.015
45. Gordon CS, Jarman HK, Rodgers RF, et al. Outcomes of a cluster randomized controlled trial of the SoMe social media literacy program for improving body image-related outcomes in adolescent boys and girls. *Nutrients* 2021; **13**(11): 3825. doi:10.3390/nu13113825
46. Gradinger P, Yanagida T, Strohmeier D, Spiel C. Effectiveness and sustainability of the ViSC Social Competence Program to prevent cyberbullying and cyber-victimization: class and individual level moderators. *Aggress Behav* 2016; **42**(2): 181–93. doi:10.1002/ab.21631
47. Gui M, Gerosa T, Argentin G, Losi L. Mobile media education as a tool to reduce problematic smartphone use: results of a randomised impact evaluation. *Comput Educ* 2023; **194**: 104705. doi:10.1016/j.compedu.2022.104705
48. Han ES, Park Y, Yurgelun-Todd D, Renshaw PF, Han DH. Comparing the effectiveness of game literacy education and game coding education in improving problematic internet gaming. *Front Psychiatry* 2024;15:1377231. doi:10.3389/fpsyt.2024.1377231
49. Hernan CJ, Collins TA, Morrison JQ, Kroeger SD. Decreasing inappropriate use of mobile devices in urban high school classrooms: comparing an antecedent intervention with and without the good behavior game. *Behav Modif* 2019;43(3):439-463. doi:10.1177/0145445518764343
50. Intolo P, Sitthikornpaiboon R, Chararivivot V. Pain and electromyography reduction cause of sitting upright posture, frequent break, eye rest and self-stretching during smartphone usage. *Work*. 2022;71(3):729-738. doi:10.3233/wor-210825
51. Jiow HJ, Mwagwabi F, Low-Lim A. Effectiveness of protection motivation theory based: password hygiene training programme for youth media literacy education. *J Media Lit Educ* 2021; **13**(1): 67–78. doi:10.23860/jmle-2021-13-1-6
52. Kapitány-Fövény M, Lukács JÁ, Takács J, et al. Gender-specific pathways regarding the outcomes of a cyberbullying youth education program. *Personality and Individual Differences* 2022;186:111338. doi: 10.1016/j.paid.2021.111338
53. Krossbakken E, Torsheim T, Mentzoni RA, et al. The effectiveness of a parental guide for prevention of problematic video gaming in children: a public health randomized controlled intervention study. *J Behav Addict* 2018; **7**(1): 52–61. doi:10.1556/2006.6.2017.087
54. Leme AC, Lubans DR, Guerra PH, Dewar D, Toassa EC, Philippi ST. Preventing obesity among Brazilian adolescent girls: six-month outcomes of the Healthy Habits, Healthy Girls-Brazil school-based randomized controlled trial. *Prev Med* 2016; **86**: 77–83. doi:10.1016/j.ypmed.2016.01.020
55. Li R, Shi G, Ji J, et al. A 2-year longitudinal psychological intervention study on the prevention of internet addiction in junior high school students of Jinan city. *Biomedical Research (India)* 2017;28:10033-10038.

56. Lubans DR, Smith JJ, Morgan PJ, et al. Mediators of psychological well-being in adolescent boys. *Journal of Adolescent Health* 2016;58(2):230-236. doi:10.1016/j.jadohealth.2015.10.010
57. Lubans DR, Smith JJ, Plotnikoff RC, et al. Assessing the sustained impact of a school-based obesity prevention program for adolescent boys: the ATLAS cluster randomized controlled trial. *International Journal of Behavioral Nutrition and Physical Activity* 2016;13(1) doi:10.1186/s12966-016-0420-8
58. Lubans DR, Smith JJ, Skinner G, Morgan PJ. Development and implementation of a smartphone application to promote physical activity and reduce screen-time in adolescent boys. *Front Public Health* 2014; **2**: 42. doi:10.3389/fpubh.2014.00042
59. Mahajan A, Negi PC, Gandhi S, Sharma D, Grover N. Impact of school-based health behavioral intervention on awareness, practice pattern of healthy lifestyle, and cardiometabolic risk factors among school children of Shimla: a cluster-randomized, intervention study. *Indian J Pediatr* 2022; **89**(4): 343–50. doi:10.1007/s12098-021-03786-6
60. Majumdar D, Koch PA, Lee H, Contento IR, Islas-Ramos ADL, Fu D. “Creature-101”: a serious game to promote energy balance-related behaviors among middle school adolescents. *Games Health J* 2013; **2**(5): 280–90. doi:10.1089/g4h.2013.0045
61. Manwong M, Lohsoonthorn V, Booranasuksakul T, Chaikoolvatana A. Effects of a group activity-based motivational enhancement therapy program on social media addictive behaviors among junior high school students in Thailand: a cluster randomized trial. *Psychol Res Behav Manag* 2018;11:329-339. doi:10.2147/prbm.S168869
62. McLean SA, Wertheim EH, Masters J, Paxton SJ. A pilot evaluation of a social media literacy intervention to reduce risk factors for eating disorders. *International Journal of Eating Disorders* 2017;50(7):847-851. doi:10.1002/eat.22708
63. Moreno MA, Klein JD, Kaseeska K, et al. A cluster randomized controlled trial of a primary care provider-delivered social media counseling intervention. *J Adolesc Health* 2023;73(5):924-930. doi:10.1016/j.jadohealth.2023.06.007
64. Mutz M, Müller J, Göring A. Outdoor adventures and adolescents’ mental health: daily screen time as a moderator of changes. *J Adventure Educ Out (JAEOL)* 2019; **19**(1): 56–66. doi:10.1080/14729679.2018.1507830
65. Nollen NL, Mayo MS, Carlson SE, Rapoff MA, Goggin KJ, Ellerbeck EF. Mobile technology for obesity prevention: a randomized pilot study in racial- and ethnic-minority girls. *Am J Prev Med* 2014; **46**(4): 404–8. doi:10.1016/j.amepre.2013.12.011
66. O’Dean S, Sunderland M, Newton N, et al. The Health4Life e-health intervention for modifying lifestyle risk behaviours of adolescents: secondary outcomes of a cluster randomised controlled trial. *Med J Aust* 2024; **220**(8): 417–24. doi:10.5694/mja2.52279
67. Ortega-Barón J, González-Cabrera J, Machimbarrena JM, Montiel I. Safety.Net: A pilot study on a multi-risk internet prevention program. *Int J Environ Res Public Health* 2021;18(8) doi:10.3390/ijerph18084249
68. Otsuka Y, Kaneita Y, Itani O, Matsumoto Y. A school-based program for problematic internet use for adolescents in Japan. *Children*. 2023; **10**(11): 1754. doi:10.3390/children10111754
69. Paiement K, Drapeau V, Gilbert JA, et al. Changes in lifestyle habits among adolescent girls after FitSpirit participation. *Int J Environ Res Public Health* 2020;17(12) doi:10.3390/ijerph17124388
70. Pietsch B, Arnaud N, Lochbühler K, et al. Effects of an app-based intervention program to reduce substance use, gambling, and digital media use in adolescents and young adults: a multicenter, cluster-randomized controlled trial in vocational schools in Germany. *Int J Environ Res Public Health* 2023;20(3) doi:10.3390/ijerph20031970
71. Rosenkranz RR, Dixon PM, Dzewaltowski DA, et al. A cluster-randomized trial comparing two SWITCH implementation support strategies for school wellness intervention effectiveness. *Journal of Sport and Health Science* 2023;12(1):87-96. doi:10.1016/j.jshs.2021.12.001
72. Scull TM, Dodson CV, Geller JG, Reeder LC, Stump KN. A Media literacy education approach to high school sexual health education: immediate effects of media aware on adolescents’ media, sexual health, and communication outcomes. *Journal of Youth and Adolescence* 2022;51(4):708-723. doi:10.1007/s10964-021-01567-0

73. Scull TM, Kupersmidt JB, Malik CV, Keefe EM. Examining the efficacy of an mHealth media literacy education program for sexual health promotion in older adolescents attending community college. *J Am Coll Health* 2018; **66**(3): 165–77. doi:10.1080/07448481.2017.1393822
74. Sevil J, García-González L, Abós Á, Generelo E, Aibar A. Can high schools be an effective setting to promote healthy lifestyles? effects of a multiple behavior change intervention in adolescents. *J Adolesc Health* 2019;64(4):478-486. doi:10.1016/j.jadohealth.2018.09.027
75. Sharma B, Kim HY, Nam EW. Effects of school-based health promotion intervention on health behaviors among school adolescents in North Lima and Callao, Peru. *J Lifestyle Med*. 2018;8(2):60-71. doi:10.15280/jlm.2018.8.2.60
76. Sheaffer J, Clarion University of P, Edinboro University of P. *Improving Parental Monitoring of Social Media Behaviors to Reduce Negative Health Outcomes*. [Clarion University of Pennsylvania] ; [Edinboro University of Pennsylvania]; 2020.
77. Shrewsbury VA, Venchiarutti RL, Hardy LL, et al. Impact and cost of the peer-led Students As LifeStyle Activists programme in high schools. *Health Education Journal* 2019;79(1):3-20. doi:10.1177/0017896919856050
78. Simons M, Brug J, Chinapaw MJM, de Boer M, Seidell J, de Vet E. Replacing non-active video gaming by active video gaming to prevent excessive weight gain in adolescents. *PLoS One* 2015; **10**(7): e0126023. doi:10.1371/journal.pone.0126023
79. Sinha A, Khatri V, Nath B. Relevance of health education to e-learning-associated problems among the school-going adolescents in Bareilly city: an interventional study. *J Family Med Prim Care* 2022;11(11):6863-6868. doi:10.4103/jfmpc.jfmpc_794_22
80. Smith JJ, Morgan PJ, Lonsdale C, Dally K, Plotnikoff RC, Lubans DR. Mediators of change in screen-time in a school-based intervention for adolescent boys: findings from the ATLAS cluster randomized controlled trial. *J Behav Med* 2017;40(3):423-433. doi:10.1007/s10865-016-9810-2
81. Smith JJ, Morgan PJ, Plotnikoff RC, et al. Smart-phone obesity prevention trial for adolescent boys in low-income communities: the ATLAS RCT. *Pediatrics* 014;134(3):e723-31. doi:10.1542/peds.2014-1012
82. Sorrentino A, Baldry AC, Farrington DP. The Efficacy of the tabby improved prevention and intervention program in reducing cyberbullying and cybervictimization among students. *International Journal of Environmental Research and Public Health* 2018;15(11):2536. doi:10.3390/ijerph15112536
83. Tamboer SL, Vlaanderen A, Bevelander KE, Kleemans M. Do you know what fake news is? An exploration of and intervention to increase youth's fake news literacy. *Youth Soc* 2024; **56**(4): 774–92. doi:10.1177/0044118x2312059300
84. Therriault D, Lane J, Houle A-A, et al. Effects of the HORS-PISTE universal anxiety prevention program measured according to initial level of student problems. *Psychology in the Schools* 2023;60(4):1299-1318. doi:doi.org/10.1002/pits.22836
85. Tsimtsiou Z, Drosos E, Drontsos A, et al. Raising awareness on cyber safety: adolescents' experience of a primary healthcare professional-led, school-based, multi-center intervention. *Int J Adolesc Med Health* 2017; **31**(6). doi:10.1515/ijamh-2017-0072
86. Tsimtsiou Z, Haidich AB, Drontsos A, Dardavesis T, Nanos P, Arvanitidou M. Exploring primary healthcare professionals' experiences as educators on safe internet use: a school-based intervention from Greece. *Rural Remote Health* 2019; **19**(2); 4806. doi:10.22605/rrh4806
87. Van Dongen B, Finn T, Hansen V, Wagemakers A, Lubans D, Dally K. The ATLAS school-based health promotion programme. *European Physical Education Review* 2018;24(3):330-348. doi:10.1177/1356336x17695834
88. Walther B, Hanewinkel R, Morgenstern M. Effects of a brief school-based media literacy intervention on digital media use in adolescents: cluster randomized controlled trial. *Cyberpsychol Behav Soc Netw* 2014;17(9):616-23. doi:10.1089/cyber.2014.0173
89. Weaver JL, Swank JM. A Mindfulness-based intervention for adolescent social media users: a quasi-experimental study. *Journal of Child and Adolescent Counseling* 2024;10(1):3-14. doi:10.1080/23727810.2023.2266895

90. White K, Lubans DR, Eather N. Feasibility and preliminary efficacy of a school-based health and well-being program for adolescent girls. *Pilot Feasibility Stud* 2022; **8**(1): 15. doi:10.1186/s40814-021-00964-3
91. Wilksch SM, Paxton SJ, Byrne SM, et al. Prevention across the spectrum: a randomized controlled trial of three programs to reduce risk factors for both eating disorders and obesity. *Psychol Med* 2015; **45**(9): 1811–23. doi:10.1017/s003329171400289x
92. Yang S-Y, Kim H-S. Effects of a prevention program for internet addiction among middle school students in South Korea. *Public Health Nurs* 2018; **35**: 246–55. doi:10.1111/phn.12394.
93. Zeichner O. The impact of safe internet intervention programs on pupils. *Journal of Educational Technology* 2019;16(3):34-43.

**Supplementary material 3. Function of intervention used in the included studies**

| First author last name | Year | Education | Persuation | Incentivisation | Coercion | Training | Restriction | Environmental restructing | Modelling | Enablement | Total |
|---|---|---|---|---|---|---|---|---|---|---|---|
| Aceves-Martin | 2022 | ☑ | ☐ | ☐ | ☐ | ☑ | ☐ | ☐ | ☐ | ☐ | 2 |
| Ahmed | 2021 | ☑ | ☐ | ☐ | ☐ | ☐ | ☐ | ☑ | ☐ | ☐ | 2 |
| Aittasalo | 2019 | ☑ | ☐ | ☐ | ☐ | ☐ | ☐ | ☐ | ☐ | ☐ | 1 |
| Aizenkot | 2018 | ☑ | ☐ | ☐ | ☐ | ☑ | ☐ | ☐ | ☐ | ☐ | 2 |
| Albert | 2019 | ☐ | ☐ | ☐ | ☐ | ☐ | ☐ | ☑ | ☐ | ☐ | 1 |
| Alert | 2015 | ☑ | ☐ | ☐ | ☐ | ☐ | ☐ | ☐ | ☐ | ☐ | 1 |
| Alle | 2017 | ☑ | ☑ | ☐ | ☐ | ☐ | ☐ | ☐ | ☐ | ☐ | 2 |
| Andrade | 2014 | ☑ | ☐ | ☐ | ☐ | ☐ | ☐ | ☑ | ☐ | ☐ | 2 |
| Andrade | 2015 | ☑ | ☐ | ☐ | ☐ | ☐ | ☐ | ☑ | ☐ | ☐ | 2 |
| Appel | 2014 | ☐ | ☐ | ☐ | ☐ | ☐ | ☐ | ☐ | ☐ | ☑ | 1 |
| Ariyadassa | 2022 | ☑ | ☐ | ☐ | ☐ | ☐ | ☐ | ☐ | ☐ | ☐ | 1 |
| Athanasiades | 2018 | ☑ | ☑ | ☐ | ☐ | ☐ | ☐ | ☐ | ☐ | ☐ | 2 |
| Austin | 2018 | ☑ | ☐ | ☐ | ☐ | ☐ | ☐ | ☐ | ☐ | ☐ | 1 |
| Avci | 2023 | ☑ | ☐ | ☐ | ☐ | ☐ | ☐ | ☐ | ☐ | ☐ | 1 |
| Babic | 2016 | ☑ | ☐ | ☐ | ☐ | ☐ | ☐ | ☐ | ☐ | ☑ | 2 |
| Ball | 2015 | ☑ | ☐ | ☐ | ☐ | ☐ | ☐ | ☑ | ☐ | ☐ | 2 |
| Bandeira | 2020 | ☑ | ☐ | ☐ | ☐ | ☑ | ☐ | ☑ | ☐ | ☐ | 3 |
| Barbosa Filho | 2019 | ☑ | ☐ | ☐ | ☐ | ☑ | ☐ | ☑ | ☐ | ☐ | 3 |
| Foley | **2017** | ☑ | ☐ | ☐ | ☐ | ☑ | ☐ | ☐ | ☐ | ☐ | **2** |
| Bickham | 2018 | ☑ | ☐ | ☑ | ☐ | ☐ | ☐ | ☑ | ☐ | ☐ | 3 |
| Bonnaire | 2019 | ☐ | ☐ | ☐ | ☐ | ☑ | ☐ | ☐ | ☐ | ☐ | 1 |
| Boor Boor | 2021 | ☐ | ☐ | ☐ | ☐ | ☑ | ☐ | ☐ | ☐ | ☐ | 1 |
| Brown | 2023 | ☑ | ☐ | ☐ | ☐ | ☐ | ☐ | ☐ | ☐ | ☑ | 2 |
| Busch | 2015 | ☑ | ☐ | ☐ | ☐ | ☑ | ☐ | ☑ | ☐ | ☐ | 3 |
| Busch | 2013 | ☑ | ☐ | ☐ | ☐ | ☑ | ☐ | ☑ | ☐ | ☐ | 3 |
| Catenacci | 2014 | ☐ | ☐ | ☐ | ☐ | ☐ | ☐ | ☑ | ☐ | ☑ | 2 |
| Cavallini | 2023 | ☐ | ☐ | ☐ | ☐ | ☑ | ☐ | ☐ | ☐ | ☑ | 2 |
| Çelik | 2016 | ☐ | ☐ | ☐ | ☐ | ☑ | ☐ | ☐ | ☐ | ☐ | 1 |
| Champion | 2023 | ☑ | ☐ | ☐ | ☐ | ☐ | ☐ | ☐ | ☐ | ☐ | 1 |
| Chau | 2019 | ☑ | ☑ | ☐ | ☐ | ☐ | ☐ | ☐ | ☐ | ☐ | 2 |

| | | | | | | | | | | | |
|---|---|---|---|---|---|---|---|---|---|---|---|
| Choi | 2020 | ☑ | ☐ | ☐ | ☐ | ☐ | ☐ | ☐ | ☐ | ☑ | 2 |
| Costa | 2018 | ☐ | ☐ | ☐ | ☐ | ☐ | ☐ | ☐ | ☐ | ☑ | 1 |
| Medina | 2018 | ☑ | ☐ | ☐ | ☐ | ☐ | ☐ | ☐ | ☐ | ☑ | 2 |
| DelRey | 2016 | ☐ | ☐ | ☐ | ☐ | ☐ | ☐ | ☐ | ☐ | ☑ | 1 |
| DelRey | 2018 | ☑ | ☐ | ☐ | ☐ | ☐ | ☐ | ☐ | ☐ | ☑ | 2 |
| Dewar | 2013 | ☑ | ☐ | ☐ | ☐ | ☐ | ☐ | ☑ | ☐ | ☐ | 2 |
| Dos Santos | 2021 | ☑ | ☐ | ☐ | ☐ | ☑ | ☐ | ☑ | ☐ | ☐ | 3 |
| Draper | 2015 | ☑ | ☐ | ☐ | ☐ | ☐ | ☐ | ☐ | ☐ | ☑ | 2 |
| Eagle | 2013 | ☑ | ☐ | ☐ | ☐ | ☐ | ☐ | ☑ | ☐ | ☐ | 2 |
| El Rayess | 2017 | ☑ | ☐ | ☐ | ☐ | ☑ | ☐ | ☐ | ☐ | ☐ | 2 |
| Fantini | 2024 | ☑ | ☐ | ☐ | ☐ | ☐ | ☐ | ☐ | ☐ | ☑ | 2 |
| Franckle | 2017 | ☑ | ☐ | ☐ | ☐ | ☐ | ☐ | ☐ | ☐ | ☑ | 2 |
| Gauld | 2021 | ☐ | ☐ | ☐ | ☐ | ☐ | ☐ | ☐ | ☐ | ☑ | 1 |
| Gordon | 2021 | ☐ | ☐ | ☐ | ☐ | ☑ | ☐ | ☐ | ☐ | ☐ | 1 |
| Gradinger | 2016 | ☐ | ☐ | ☐ | ☐ | ☑ | ☐ | ☐ | ☐ | ☐ | 1 |
| Gui | 2023 | ☐ | ☐ | ☐ | ☐ | ☑ | ☐ | ☐ | ☐ | ☐ | 1 |
| Han | 2024 | ☑ | ☐ | ☐ | ☐ | ☐ | ☐ | ☐ | ☐ | ☐ | 1 |
| Hernan | 2019 | ☐ | ☐ | ☑ | ☐ | ☐ | ☑ | ☑ | ☐ | ☐ | 3 |
| Intolo | 2022 | ☑ | ☐ | ☐ | ☐ | ☐ | ☐ | ☐ | ☐ | ☐ | 1 |
| Jiow | 2021 | ☑ | ☐ | ☐ | ☐ | ☐ | ☐ | ☐ | ☐ | ☐ | 1 |
| Weaver | 2024 | ☐ | ☐ | ☐ | ☐ | ☐ | ☐ | ☐ | ☐ | ☑ | 1 |
| Kapitany-Fövény | 2022 | ☑ | ☐ | ☐ | ☐ | ☐ | ☐ | ☐ | ☐ | ☑ | 2 |
| Krossbakken | 2017 | ☑ | ☐ | ☐ | ☐ | ☐ | ☐ | ☐ | ☐ | ☐ | 1 |
| Leme | 2016 | ☑ | ☐ | ☐ | ☐ | ☐ | ☐ | ☐ | ☐ | ☑ | 2 |
| Li | 2017 | ☑ | ☐ | ☐ | ☐ | ☐ | ☐ | ☐ | ☐ | ☐ | 1 |
| Lubans | 2014 | ☑ | ☐ | ☐ | ☐ | ☑ | ☐ | ☑ | ☐ | ☐ | 3 |
| Lubans | 2016 | ☑ | ☐ | ☐ | ☐ | ☑ | ☐ | ☑ | ☐ | ☐ | 3 |
| Lubans | 2016 | ☑ | ☐ | ☐ | ☐ | ☑ | ☐ | ☑ | ☐ | ☐ | 3 |
| Mahajan | 2022 | ☑ | ☐ | ☐ | ☐ | ☑ | ☐ | ☑ | ☐ | ☐ | 3 |
| Majumdar | 2013 | ☑ | ☐ | ☐ | ☐ | ☑ | ☐ | ☐ | ☐ | ☑ | 3 |
| Manwong | 2018 | ☑ | ☐ | ☐ | ☐ | ☐ | ☐ | ☐ | ☐ | ☑ | 2 |
| McLean | 2017 | ☑ | ☐ | ☐ | ☐ | ☐ | ☐ | ☐ | ☐ | ☐ | 1 |
| Moreno | 2023 | ☑ | ☐ | ☐ | ☐ | ☑ | ☐ | ☐ | ☐ | ☐ | 2 |

| | | | | | | | | | | | |
|---|---|---|---|---|---|---|---|---|---|---|---|
| Mutz | 2019 | ☑ | ☐ | ☐ | ☐ | ☐ | ☐ | ☑ | ☐ | ☐ | 2 |
| Nollen | 2014 | ☑ | ☐ | ☐ | ☐ | ☐ | ☐ | ☐ | ☐ | ☑ | 2 |
| O'Dean | 2024 | ☑ | ☑ | ☐ | ☐ | ☐ | ☐ | ☐ | ☐ | ☐ | 2 |
| Ortega-Baron | 2021 | ☑ | ☐ | ☐ | ☐ | ☐ | ☐ | ☐ | ☐ | ☐ | 1 |
| Otsuka | 2023 | ☑ | ☐ | ☐ | ☐ | ☐ | ☐ | ☐ | ☐ | ☐ | 1 |
| Paiement | 2020 | ☑ | ☐ | ☐ | ☐ | ☐ | ☐ | ☑ | ☐ | ☐ | 2 |
| Balhara | 2019 | ☑ | ☐ | ☐ | ☐ | ☐ | ☐ | ☐ | ☐ | ☐ | 1 |
| Pietsh | 2023 | ☐ | ☐ | ☐ | ☐ | ☐ | ☐ | ☐ | ☐ | ☑ | 1 |
| Rosenkrank | 2021 | ☐ | ☐ | ☐ | ☐ | ☐ | ☐ | ☐ | ☐ | ☑ | 1 |
| Tamboer | 2024 | ☑ | ☐ | ☐ | ☐ | ☐ | ☐ | ☐ | ☐ | ☐ | 1 |
| Scull | 2018 | ☑ | ☐ | ☐ | ☐ | ☐ | ☐ | ☐ | ☐ | ☐ | 1 |
| Scull | 2014 | ☑ | ☐ | ☐ | ☐ | ☑ | ☐ | ☐ | ☐ | ☐ | 2 |
| Sevil | 2019 | ☐ | ☐ | ☐ | ☐ | ☐ | ☐ | ☑ | ☐ | ☑ | 2 |
| Sharma | 2018 | ☑ | ☐ | ☐ | ☐ | ☐ | ☐ | ☑ | ☐ | ☐ | 2 |
| Sheaffer | 2020 | ☑ | ☐ | ☐ | ☐ | ☑ | ☐ | ☐ | ☐ | ☐ | 2 |
| Shrewsburry | 2020 | ☑ | ☐ | ☐ | ☐ | ☑ | ☐ | ☐ | ☐ | ☐ | 2 |
| Sinha | 2022 | ☑ | ☐ | ☐ | ☐ | ☑ | ☐ | ☐ | ☐ | ☐ | 2 |
| Simons | 2015 | ☐ | ☐ | ☐ | ☐ | ☐ | ☐ | ☐ | ☐ | ☑ | 1 |
| Sorrentino | 2018 | ☑ | ☐ | ☐ | ☐ | ☑ | ☐ | ☐ | ☐ | ☐ | 2 |
| Smith | 2017 | ☑ | ☐ | ☐ | ☐ | ☑ | ☐ | ☑ | ☐ | ☐ | 3 |
| Smith | 2014 | ☑ | ☐ | ☐ | ☐ | ☑ | ☐ | ☑ | ☐ | ☐ | 3 |
| Therriault | 2022 | ☑ | ☐ | ☐ | ☐ | ☐ | ☐ | ☐ | ☐ | ☑ | 2 |
| Tsimtsiou | 2017 | ☑ | ☐ | ☐ | ☐ | ☐ | ☐ | ☐ | ☐ | ☐ | 1 |
| Tsimtsiou | 2019 | ☑ | ☐ | ☐ | ☐ | ☑ | ☐ | ☐ | ☐ | ☐ | 2 |
| Van dogen | 2018 | ☑ | ☐ | ☐ | ☐ | ☑ | ☐ | ☑ | ☐ | ☐ | 3 |
| Walther | 2014 | ☑ | ☐ | ☐ | ☐ | ☐ | ☐ | ☐ | ☐ | ☑ | 2 |
| White | 2022 | ☐ | ☐ | ☐ | ☐ | ☐ | ☐ | ☐ | ☐ | ☑ | 1 |
| Wilksch | 2015 | ☑ | ☐ | ☐ | ☐ | ☐ | ☐ | ☐ | ☐ | ☐ | 1 |
| Yang | 2018 | ☐ | ☐ | ☐ | ☐ | ☐ | ☐ | ☐ | ☑ | ☑ | 2 |
| Zeichner | 2019 | ☑ | ☐ | ☐ | ☐ | ☐ | ☐ | ☐ | ☐ | ☐ | 1 |
| Total | | 72 | 4 | 2 | 0 | 31 | 1 | 26 | 1 | 28 | |
| **%** | | **78%** | **4%** | **2%** | **0%** | **34%** | **1%** | **28%** | **1%** | **30%** | |
