# Supplementary material for: Scoping review of interventions aimed at promoting healthy screen use among adolescents
Source: BMJ Open. 2025 Sep 21;15(9):e103772. doi: 10.1136/bmjopen-2025-103772 (PMC12458889; doi:10.1136/bmjopen-2025-103772)
Supplement: Supplementary file 2 [file bmjopen-2025-103772.reviewer_comments.pdf]

PEER REVIEW HISTORY

BMJ Open publishes all reviews undertaken for accepted manuscripts. Reviewers are asked to complete a checklist review form and are provided with free text boxes to elaborate on their assessment. These free text comments are reproduced below.

ARTICLE DETAILS

Title (Provisional)

A Scoping Review of Interventions Aimed at Promoting Healthy Screen Use Among Adolescents

Authors

Tock, Wing Lam; Gauvin, Lise; Hudon, Frédérique; Tremblay, Frédérique; Bélanger, Richard E; Turcotte-Tremblay, Anne-Marie

---

VERSION 1 - REVIEW

**Reviewer**	**1**
**Name**	**Raeside, Rebecca**
**Affiliation**	**University of Sydney, Susan Wakil School of Nursing and Midwifery**
**Date**	**04-Jun-2025**
**COI**	**None**

Thank you for the opportunity to review this scoping review of interventions aimed at promoting healthy screen use among adolescents. This review identified 93 articles, representing 81 interventions. I commend the authors for a thorough review, adhering to Arksey and O'Malley and PRISMA-ScR guidelines. Nonetheless, I have a few comments to strengthen the manuscript prior to publication:

Abstract:

- The aim of this scoping review isn't clear from the abstract, please revise
- More details on scoping review methodology are needed in the abstract, please revise

Introduction:

- As it currently reads, the introduction is focused mostly on the negative effects of screen use. It would be helpful for the reader if there was more of a balance with evidence related to benefits of screen use among adolescents and a definition of 'healthy screen use' presented in the introduction.

Methods:

- Interventions targeting parents, educators, health professionals, law enforcement were also included – what is the rationale for this?

Results:

- Age – it would be helpful if you are clear in your inclusion criteria on including studies where only some of the participants would fall inside the 10-19 age range, or whether you used the mean age to assess suitability for inclusion? Upon looking at Figure 3, it seems you have included studies where most participants would fall outside 10-19 years e.g. Intolo, 2022
- What is the total number of participants included with all included studies? Was a mean age able to be calculated?
- Outcomes – I am unsure why studies that target outcomes including 'sexual health' or 'password setting' would fall within the inclusion criteria for this study. Can you please provide further context as to why these studies are included?
- What is the total number of interventions with positive intervention effects on healthy screen use?
- 'Interventions that targeted multiple health behaviours showed effectiveness in reorienting adolescents from screen time to alternative health behaviours, such as physical activity'. How many interventions demonstrated this? Putting numerical values in here may help to demonstrate the strength of the evidence.

Discussion:

- It would be great to see some more discussion relating to reorienting adolescents to alternative health behaviours. Does this require multiple intervention functions to work in parallel? What can be learnt from those which were successful?
- Though only a small number of studies, I cannot determine how the qualitative results have impacted the overall study findings. Were there any interesting findings from these studies and how were they incorporated into this review?
- I agree with your proposed future directions for intervention and research. One of your proposed future directions includes engaging with adolescents in designing and implementing interventions. I wonder whether this can be expanded on in the discussion. Did any of the included studies work with adolescents to develop the intervention(s)? What was the impact of this?

Other comments:

Figure 4: I wonder whether data was collected on the number of studies which employed these specific intervention functions, can this be added?

**Reviewer**	**2**
**Name**	**Ferrari, Manuela**
**Affiliation**	**McGill University**
**Date**	**25-Jun-2025**
**COI**	**None**

Review of Manuscript: “A Scoping Review of Interventions Aimed at Promoting Healthy Screen Use Among Adolescents”

Submitted to: BMJ Open

It was a pleasure to be invited to review this manuscript. I commend the authors for undertaking a timely and highly relevant scoping review. The topic of adolescent screen use continues to gain urgency as digital technologies become ever more integrated into adolescents’ daily lives. This work has the potential to make a meaningful contribution to the current literature and to inform public health interventions, education, and policy.

I recommend revisions to strengthen the manuscript further. Below, I outline suggestions organized by key areas:

1. Introduction

I was pleased to see that the authors have adopted a public health framework. I encourage you to further elaborate on how social determinants of health influence adolescent screen use. For example, factors such as neighborhood safety, access to outdoor recreational spaces, and availability of after-school programming can shape adolescents’ screen-related behaviors. Expanding on this would help establish a stronger link between your introduction, results, and discussion.

2. Literature Contextualization

I suggest incorporating the following commentary, which may add useful theoretical depth:

Ferrari, M., & Schick, A. (2020). Teenagers, screens and social media: a commentary on Orben’s narrative review. Social Psychiatry and Psychiatric Epidemiology, 55(8), 973–975.

This piece explores the complexity of adolescent screen use, including the importance of content type, context, and individual-level differences—dimensions that could enrich the interpretation of your results.

3. Limitations and Co-Design

Please consider emphasizing the need for adolescent participation in the design and implementation of interventions. Both the "Strengths and limitations of this study: table" and discussion section would benefit from explicit mention of the importance of co-designing strategies with adolescents to improve engagement, cultural relevance, and impact -see also next Table 2 Future Directions.

4. Language Scope of Review

It is commendable that studies published in all languages were included. However, please clarify how non-English studies were handled—e.g., how they were screened, translated (if applicable), and reviewed. This is important for assessing the inclusivity and rigor of your methodology.

5. Data Consistency

There are inconsistencies in the reporting of data across the manuscript. Please ensure that both n and % are consistently reported in all relevant sections, including tables and narrative summaries.

6. Balanced Interpretation of Evidence

Some statements would benefit from a more balanced presentation of the evidence. For example, the assertion that gaming is associated with aggression and cyberbullying should be accompanied by research indicating potential cognitive, attentional, and prosocial benefits of gaming. This will help avoid one-sided interpretations and reflect the nuanced state of the literature.

7. Table 2 – Future Directions

Consider adding the need for participatory and co-designed approaches with adolescents as a clear future direction in Table 2. Adolescent engagement is increasingly seen as essential in intervention science, and its inclusion would align well with your broader equity-focused framing.

8. Equity and Subgroup Considerations

The section “Considerations for equity or differing effects based on population subgroups” would be strengthened by more detailed reporting on the adolescent populations involved in the studies. For instance, were equity-deserving groups (e.g., racialized adolescents, those with disabilities, LGBTQ+ adolescents), rural/urban distinctions, or socioeconomic status (SES) characteristics explicitly described? This would help assess the generalizability and relevance of the interventions reviewed.

9. Policy Context

Your discussion around the limitations of restrictive screen use policies is very thoughtful. You mention a specific policy that was abolished due to human rights and efficacy concerns. I wonder whether the lack of identified effective policy interventions could be due to the search strategy and terminology used. A brief reflection on this possibility would be a valuable addition to the limitations section.

Conclusion

Overall, this manuscript is well-structured, relevant, and offers an important synthesis of existing interventions targeting adolescent screen use. With these revisions, the paper will be well-positioned to inform both academic audiences and those involved in the design of adolescent-centered health strategies.

Thank you for the opportunity to review this important work.

VERSION 1 - AUTHOR RESPONSE

Reviewer: 1
Comments to the Author:

Thank you for the opportunity to review this scoping review of interventions aimed at promoting healthy screen use among adolescents. This review identified 93 articles, representing 81 interventions. I commend the authors for a thorough review, adhering to Arksey and O'Malley and PRISMA-ScR guidelines. Nonetheless, I have a few comments to strengthen the manuscript prior to publication:

Abstract:

- The aim of this scoping review isn't clear from the abstract, please revise

- More details on scoping review methodology are needed in the abstract, please revise

Thank you for this helpful comment. We thoroughly revised the abstract to ensure that the aim of the scoping review is clearly articulated. We also added details on the review's methodology. Following the editor's suggestion, the abstract now follows a structured format including Objectives, Design, Data Sources, Eligibility Criteria, Data Extraction and Synthesis, Results, and Conclusions. These revisions enhance the clarity of the abstract section.

Introduction:

- As it currently reads, the introduction is focused mostly on the negative effects of screen use. It would be helpful for the reader if there was more of a balance with evidence related to benefits of screen use among adolescents and a definition of 'healthy screen use' presented in the introduction.

Thank you for this insightful comment. We revised the introduction to incorporate a more balanced perspective by including evidence on the potential benefits of screen use among adolescents. For example, we now highlight how some digital media and screens can facilitate social connections and support learning. (p. 4, lines 68-71).

Regarding the definition of "healthy screen use", we agree that conceptual clarity is important for advancing the field. However, as noted in the literature, this remains an emerging area with no universally accepted conceptual definition. Concepts such as "digital wellness," "balanced screen use," and "healthy media" are used inconsistently across studies. We have added this observation to the introduction, along with a brief synthesis of how the term is variably conceptualized (p. 5, lines 114-119). Additionally, in our discussion, "recommendation for future research" section, we further emphasize the need for greater conceptual clarity and consensus among researchers and practitioners to support the development, implementation, and evaluation of interventions in this area.

Methods:

- Interventions targeting parents, educators, health professionals, law enforcement were also included – what is the rationale for this?

Thank you for this question. All interventions included in this review are interventions aimed at **promoting healthy screen use among adolescents**. Some of these interventions **target guardians or caregivers of adolescents** including parents, educators, health professionals, and law enforcement

as they can play a key role in helping young adolescents improve their screen use. In all of these cases, however, the interventions' ultimate goals were to influence adolescent screen behaviours. For example, the study by Krossbakken et al. (2017) evaluated a parental intervention designed to prevent problematic video game use in children aged 8 to12 years. Although the intervention targeted parents, its aim was to improve the digital health and well-being of the children and adolescents, thus aligning with the objectives of our review.

Given that this is a scoping review, our intention was to be as inclusive as possible to capture the full range of intervention strategies relevant to adolescent screen use. We modified the manuscript to clarify this rationale and we revised the inclusion criteria accordingly in the manuscript (p. 6, lines 143-145).

Results:

- Age – it would be helpful if you are clear in your inclusion criteria on including studies where only some of the participants would fall inside the 10-19 age range, or whether you used the mean age to assess suitability for inclusion? Upon looking at Figure 3, it seems you have included studies where most participants would fall outside 10-19 years e.g. Intolo, 2022

We included studies in which either all of the participants or a subsample of participants fell within the 10–19 year old age range. We included studies in which a portion of participants were within this age group only if the results were relevant and presented for this age group. We clarified this rationale and revised the inclusion criteria accordingly in the manuscript (p. 6, lines 145-147).

- What is the total number of participants included with all included studies? Was a mean age able to be calculated?

Given the heterogeneity of study populations across the included studies (i.e., adolescents, parents, child-parent dyads, educators, health professionals, and law enforcement), we have added a breakdown of participant types to provide readers with an overview of who was targeted in each study (p. 9, lines 243-248). We did not include studies that studies did not report the actual number of participants. For example, in Franckle et al (2017), they only reported numbers of communities included in the intervention, but not the actual sample size.

Regarding mean age, we were unable to calculate an overall mean age across studies, as many did not systematically report this information (sample size, age and standard deviation). Several studies provided only age ranges, school grade levels, or no age data at all. Therefore, we were unable to calculate a pooled or average mean age for adolescent participants. Given that we are not conducting a meta-analysis, we believe that this would not impact the results and conclusion of our scoping review.

- Outcomes – I am unsure why studies that target outcomes including 'sexual health' or 'password setting' would fall within the inclusion criteria for this study. Can you please provide further context as to why these studies are included?

Thank you for this important question. As outlined in our inclusion criteria, we adopted a broad conceptualization of "healthy screen use," which includes both the promotion of positive behaviors and the prevention of screen-related risks. We therefore included a range of digital behaviors that have been targeted by screen-related interventions for adolescents. For example, screen use presents specific risks related to sexting and online sexual behaviors. Some interventions have targeted these behaviors to ensure that adolescents are protected online and

equipped with the skills to assess risks, make informed decisions, and respond appropriately to online sexual content. Similarly, password setting and other cybersecurity-related behaviors were considered part of promoting safe and responsible screen use as adolescents navigate increasingly complex digital environments. Password setting is an important aspect of digital literacy. This part of the broader skill set required to navigate and protect oneself in digital environments, which falls under digital literacy. These studies align with our intention to map the full range of interventions that promote healthy screen use and prevent screen-related risks.

- What is the total number of interventions with positive intervention effects on healthy screen use?

We have now added the total number of interventions that reported positive effects on healthy screen use (p. 10, line 309). However, this information is provided for descriptive purposes only, in keeping with the objectives of a scoping review. Given the heterogeneity in study designs, outcomes, and follow-up periods, this count should not be interpreted inferentially or as an assessment of effectiveness.

- 'Interventions that targeted multiple health behaviours showed effectiveness in reorienting adolescents from screen time to alternative health behaviours, such as physical activity'. How many interventions demonstrated this? Putting numerical values in here may help to demonstrate the strength of the evidence.

We have reviewed the relevant studies and added the number of interventions that targeted multiple health behaviors and demonstrated effectiveness in reducing screen time while promoting alternative behaviors (p. 11, line 316).

Discussion:

- It would be great to see some more discussion relating to reorienting adolescents to alternative health behaviours. Does this require multiple intervention functions to work in parallel? What can be learnt from those which were successful?

We have expanded the discussion to reflect on how successful interventions reoriented adolescents from excessive screen use toward alternative health-promoting behaviors (e.g., physical activity). We now comment on the potential role of using multiple intervention functions in tandem such as education, environmental restructuring, and enablement to support this shift. We cite examples from the included studies that demonstrated positive changes in both screen-related and alternative health outcomes. These additions are now reflected in the revised discussion (p. 13, lines 371-374).

- Though only a small number of studies, I cannot determine how the qualitative results have impacted the overall study findings. Were there any interesting findings from these studies and how were they incorporated into this review?

Thank you for highlighting this point. In fact, most of the qualitative studies (and those with qualitative components) were process evaluations or feasibility studies focused on assessing participants' perceptions, acceptability, and contextual influences on intervention delivery. We have described these results in the subsection of the results section "Lessons learned from studying intervention implementation processes" (p. 11, line 334). We mentioned that although they did not directly assess intervention effectiveness, these studies provided valuable insights into the mechanisms, facilitators, and barriers to implementation.

- I agree with your proposed future directions for intervention and research. One of your proposed future directions includes engaging with adolescents in designing and implementing interventions. I wonder whether this can be expanded on in the discussion. Did any of the included studies work with adolescents to develop the intervention(s)? What was the impact of this?

As our inclusion criteria focused on studies that evaluated intervention outcomes, we did not systematically review studies focused on the intervention development process. As a result, detailed information about adolescent involvement in intervention co-design was often not reported or available. Since this is not an objective of the review, we did not mention it in the Discussion section. Nonetheless, we recognize the importance of engaging adolescents as active partners in intervention design; therefore, we recommend the use of participatory methods in future screen use interventions.

Other comments:

- Figure 4: I wonder whether data was collected on the number of studies which employed these specific intervention functions, can this be added?

Yes, we did collect and document data on the number and percentage of studies employing each of the intervention functions. These data are detailed in Supplementary Material 3, where we list each included study alongside the intervention function(s) applied. This table also includes summary counts and percentages for each function. We have now added a cross-reference to this supplementary material in the footnote for Figure 4 to clarify this for readers.

Reviewer: 2

Comments to the Author:
Review of Manuscript: “A Scoping Review of Interventions Aimed at Promoting Healthy Screen Use Among Adolescents”
Submitted to: BMJ Open

It was a pleasure to be invited to review this manuscript. I commend the authors for undertaking a timely and highly relevant scoping review. The topic of adolescent screen use continues to gain urgency as digital technologies become ever more integrated into adolescents’ daily lives. This work has the potential to make a meaningful contribution to the current literature and to inform public health interventions, education, and policy.

I recommend revisions to strengthen the manuscript further. Below, I outline suggestions organized by key areas:

1. Introduction
I was pleased to see that the authors have adopted a public health framework. I encourage you to further elaborate on how social determinants of health influence adolescent screen use. For example, factors such as neighborhood safety, access to outdoor recreational spaces, and availability of after-school programming can shape adolescents’ screen-related behaviors. Expanding on this would help establish a stronger link between your introduction, results, and discussion.

Thank you for this suggestion. We have now expanded the introduction and cited additional literature to discuss how social and environmental factors can shape adolescents' screen behaviors (page 5, lines 116-121).

2. Literature Contextualization
I suggest incorporating the following commentary, which may add useful theoretical depth:
Ferrari, M., & Schick, A. (2020). Teenagers, screens and social media: a commentary on Orben's narrative review. Social Psychiatry and Psychiatric Epidemiology, 55(8), 973–975.
This piece explores the complexity of adolescent screen use, including the importance of content type, context, and individual-level differences—dimensions that could enrich the interpretation of your results.

Thank you for this suggestion. We have incorporated this reference into the discussion to emphasize that the effects of screen use are shaped not only by duration, but also by the nature of content, contextual factors, and individual-level differences (p. 15, line 440-443).

3. Limitations and Co-Design
Please consider emphasizing the need for adolescent participation in the design and implementation of interventions. Both the "Strengths and limitations of this study: table" and discussion section would benefit from explicit mention of the importance of co-designing strategies with adolescents to improve engagement, cultural relevance, and impact -see also next Table 2 Future Directions.

Thank you for this comment. We have added a recommendation for future intervention: engage stakeholders including adolescents to enhance interventions' relevance and applicability and facilitate their integration into existing systems (see Table 2.) We do not cite this as a limitation but rather as a characteristic of the current database. Future meta-analyses may allow for comparisons demonstrating that stakeholder engagement results in more effective interventions.

4. Language Scope of Review
It is commendable that studies published in all languages were included. However, please clarify how non-English studies were handled—e.g., how they were screened, translated (if applicable), and reviewed. This is important for assessing the inclusivity and rigor of your methodology.

Thank you for highlighting this important point. As part of our inclusive search strategy, we did not apply any language restrictions. Articles in languages other than English were screened based on their English-language titles and abstracts, which were available through the databases we used. No non-English articles met the eligibility criteria for full-text screening. Also, as reported in our results, the vast majority of included interventions were conducted in English-speaking countries, particularly the United States and Australia.

5. Data Consistency
There are inconsistencies in the reporting of data across the manuscript. Please ensure that both n and % are consistently reported in all relevant sections, including tables and narrative summaries.

We have carefully reviewed the manuscript to ensure consistency in data reporting. Where applicable, both numbers (n) and corresponding percentages (%) have been added to enhance clarity for readers.

6. Balanced Interpretation of Evidence
Some statements would benefit from a more balanced presentation of the evidence. For example,

the assertion that gaming is associated with aggression and cyberbullying should be accompanied by research indicating potential cognitive, attentional, and prosocial benefits of gaming. This will help avoid one-sided interpretations and reflect the nuanced state of the literature.

Thank you for this important suggestion. In response, we have revised the relevant paragraph in the discussion to acknowledge research highlighting potential cognitive, attentional, and prosocial benefits of certain types of video gaming (p. 14, lines 435-436). This addition provides a more balanced perspective on gaming-related behaviors.

7. Table 2 – Future Directions
Consider adding the need for participatory and co-designed approaches with adolescents as a clear future direction in Table 2. Adolescent engagement is increasingly seen as essential in intervention science, and its inclusion would align well with your broader equity-focused framing.

As mentioned above, we have added the recommendation for future intervention (see Table 2, first recommendation)

8. Equity and Subgroup Considerations
The section "Considerations for equity or differing effects based on population subgroups" would be strengthened by more detailed reporting on the adolescent populations involved in the studies. For instance, were equity-deserving groups (e.g., racialized adolescents, those with disabilities, LGBTQ+ adolescents), rural/urban distinctions, or socioeconomic status (SES) characteristics explicitly described? This would help assess the generalizability and relevance of the interventions reviewed.

Thank you for your suggestion. In response, we have clarified in the manuscript that only a small number of included studies explicitly targeted or described equity-deserving groups (p. 10, line 357). Specifically, we identified two interventions conducted in low-income communities, two in rural settings, and one that focused on an ethnic minority group. Although some interventions were sex-specific, no studies in our review explicitly targeted LGBTQ+ adolescents, adolescents with disabilities, or conducted subgroup comparisons by SES, race, or gender identity.

9. Policy Context
Your discussion around the limitations of restrictive screen use policies is very thoughtful. You mention a specific policy that was abolished due to human rights and efficacy concerns. I wonder whether the lack of identified effective policy interventions could be due to the search strategy and terminology used. A brief reflection on this possibility would be a valuable addition to the limitations section.

Indeed, our search strategy focused on interventions. We did not explicitly target policies. As such, relevant policies may not have been captured in this review. We have added a note in the limitations section to reflect this point (page 16, lines 453-455). Recognizing this gap, our team is currently conducting a separate scoping review focused on policies related to adolescent screen use.
